# ASO-based PKM splice-switching therapy increases anti-CTLA-4 antibody efficacy in pancreatic ductal adenocarcinoma

**DOI:** 10.1038/s41421-026-00882-9

**Published:** 2026-04-21

**Authors:** Lijie Han, Lina Gan, Balázs Schäfer, Dillon M. Voss, Mathias Danielsen, Ondrej Kostov, Jia Liu, Ting Wang, Marvin H. Caruthers, Hao Chen, Adrian R. Krainer

**Affiliations:** 1 Cold Spring Harbor Laboratory, Cold Spring Harbor, NY USA; 2 Department of General Surgery, Pancreatic Disease Center, Ruijin Hospital, Shanghai Jiaotong University School of Medicine, Shanghai, China; 3 Department of Biochemistry, University of Colorado, Boulder, CO USA; 4 Renaissance School of Medicine, Stony Brook University, Stony Brook, NY USA; 5 Department of Pathology, Ruijin Hospital, Shanghai Jiao Tong University School of Medicine, Shanghai, China

**Keywords:** Cancer metabolism, Targeted therapies, RNA splicing

## Abstract

The alternative splice isoform of pyruvate kinase M (PKM), PKM2, plays a pivotal role in regulating aerobic glycolysis in tumor cells. Systemic delivery of antisense oligonucleotides (ASOs) that shift *PKM* splicing from the PKM2 isoform to the PKM1 isoform inhibits tumor progression and reprograms intratumoral metabolism. However, the cellular populations within the tumor microenvironment (TME) are also highly dependent on PKM2 and might likewise be affected by ASO treatment. In this study, we demonstrate that PKM2 is upregulated and PKM1 is downregulated in both human and murine pancreatic ductal adenocarcinoma (PDAC) cells. PKM1 and PKM2 are mutually exclusive and expressed in a cell type-specific manner in various cell types and stages of PDAC tumors. We report that basal-like PDAC cells and their surrounding activated regulatory T cells (T_regs_) rely on PKM2 to sustain glycolysis. Although PKM-ASO monotherapy had a limited effect in an immunodeficient mouse model of PDAC, synergy between PKM-ASO and anti-CTLA-4 immune checkpoint blockade (ICB), which targets T_regs_, restricted tumor growth in an immunocompetent mouse model. Our findings provide preclinical support for combined antisense therapy and ICB for PDAC patients, highlighting the critical role of PKM2 in the TME and its potential as a therapeutic target.

## Introduction

Pancreatic ductal adenocarcinoma (PDAC) is among the most lethal cancers worldwide, with most patients showing a limited response to immune checkpoint blockade (ICB) because of the immunosuppressive tumor microenvironment (TME)^1^. Metabolic reprogramming contributes to this distinctive TME. Specifically, terminally differentiated neutrophils, characterized by hyperactivated glycolysis, exhibit pro-tumorigenic functions in both mice and humans with PDAC^2,3^. Increased oxidative phosphorylation (OXPHOS) and glycolysis contribute to the differentiation and infiltration of T_regs_ in the TME^4–6^. Furthermore, inhibiting glycolysis and lactic acid production was reported to increase the efficacy of ICB by restricting T_reg_ function^7,8^. However, therapeutic strategies targeting these metabolic features have not reached the clinic because of their low selectivity or unexpected toxicity^9^.

Pyruvate kinase (PK), which catalyzes the conversion of phosphoenolpyruvate (PEP) to pyruvate, is a rate-limiting enzyme in glycolysis^10^. In mammals, four PK isoforms, PKL, PKR, PKM1, and PKM2, are encoded by the genes *PKLR* (PKL and PKR) and *PKM* (PKM1 and PKM2)^11^. Tissue-specific promoters drive *PKLR* transcription, with PKL predominantly expressed in the liver and kidney and PKR primarily expressed in red blood cells^11^. Most other tissues express either the PKM1 or the PKM2 isoform. PKM1 is expressed mainly in terminally differentiated, non-proliferating cells, such as muscle and nerve cells, whereas PKM2 is expressed predominantly in embryonic cells, immune cells, and the majority of cancer cells to support their metabolic demand for rapid ATP production and anabolic biosynthesis^10^. Mutually exclusive alternative splicing of *PKM* pre-mRNA exons 9 and 10 generates these two distinct PKM isoforms^12^. PKM1 is a constitutively active enzyme, whereas PKM2 has lower basal enzymatic activity but is allosterically regulated by fructose-1,6-bisphosphate (FBP)^13^. The reduced activity of PKM2 provides tumors with a proliferative advantage^14^. On the basis of these observations, PKM2 activators were designed to promote tetramer formation and were shown to suppress the tumorigenesis of non-small cell lung cancer (NSCLC) subcutaneous xenografts in mice^15^. In an alternative strategy, downregulation of PKM2 in hepatocellular carcinoma (HCC) cells abrogated tumor cell proliferation in vitro^16^. Interestingly, CRISPR-mediated knockout of PKM2-specific exon 10 in tumor-infiltrating CD8^+^ T cells synergizes with PD-1 blockade and induces long-term remission in mouse models of NSCLC and melanoma but has only a limited effect without PD-1 blockade^17^. Similarly, the PKM2 agonist TEPP46 synergizes with anti-PD1 therapy to activate effector T cells while reducing the number of T_regs_ in melanoma and colon cancer models^18^. Furthermore, overexpression of PKM1 in certain contexts can suppress tumor growth, although various studies have reported that PKM1 has either pro-tumorigenic or tumor-suppressive activity^19–21^.

We previously reported modified antisense oligonucleotides (ASOs) that switch the alternative splicing of *PKM* from PKM2 to PKM1, inhibiting HCC growth both in vitro and in vivo^22^. Systemic delivery of such splice-switching PKM-ASOs enables their uptake not only by tumor cells but also by various components within the TME^22^. Therefore, determining the role of PKM in the TME is important for the development of a PKM-ASO-based treatment. Unlike HCC, PDAC is characterized by distinct subtypes, classical and basal-like, with different metabolic modes and a dense fibrotic stroma composed of cancer-associated fibroblasts (CAFs), immunosuppressive cells, and nerve cells^23^. To some extent, systemically delivered ASOs accumulate in the normal pancreas^24^. Moreover, PKM-ASOs inhibit PDAC patient-derived organoid proliferation^25^, supporting the potential of PKM-ASO for PDAC therapy.

Here, we report that the expression patterns of PKM2 and PKM1 are cell-type specific in PDAC tumors and their TMEs. Both basal-like PDAC cells and their surrounding activated T_regs_ rely on PKM2 to sustain glycolysis. Although PKM-ASO monotherapy had a limited effect in an immunodeficient PDAC mouse model, synergy between PKM-ASOs and an anti-CTLA-4 antibody markedly inhibited tumor growth in an immunocompetent mouse model. These findings provide preclinical evidence supporting the combination of antisense therapy with ICB for PDAC patients.

## Results

### Tissue-specific expression of PKM1 and PKM2 and its clinical significance in PDAC

**Fig. 1:**
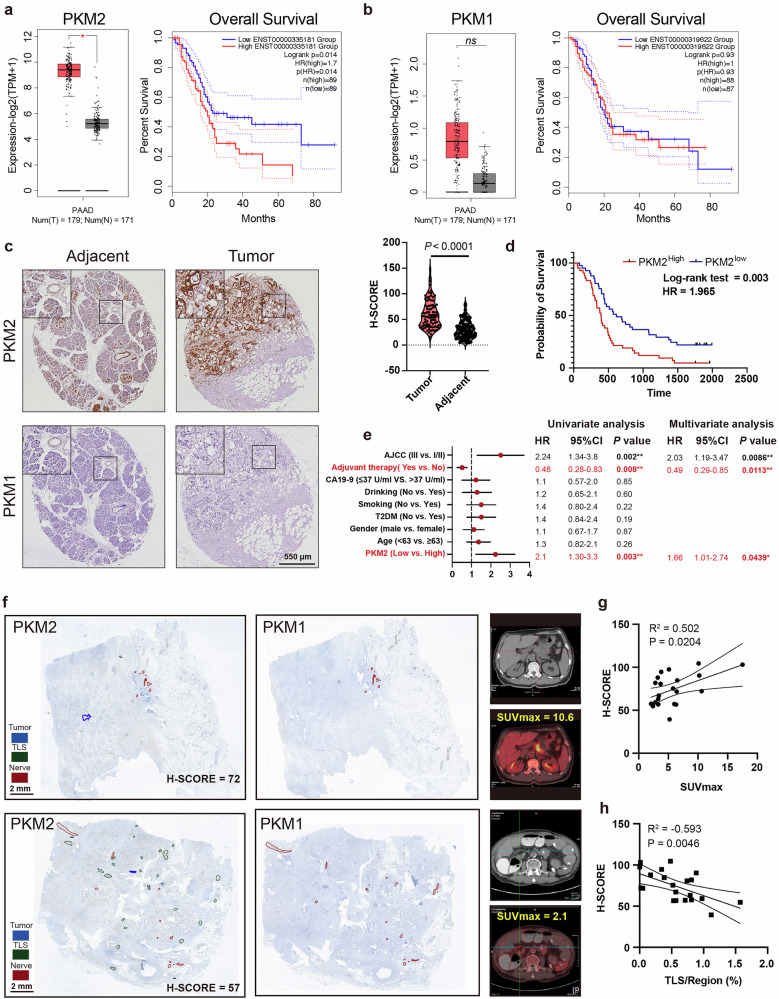
Expression and localization of PKM1/2 in the PDAC TME and clinical significance. **a**, **b** mRNA levels of PKM1 and PKM2 and Kaplan–Meier survival analysis of patients with PDAC from The Cancer Genome Atlas (TCGA). Group cutoffs were set at the median mRNA expression of PKM2 (89 patients per group) or PKM1 (High = 88, Low = 87). **c** Representative immunohistochemical staining images with anti-PKM2 and anti-PKM1 antibodies. Violin plot showing the PKM2 IHC intensity score. Scale bar, 550 μm. Images are representative of 83 PDAC samples. **d** Overall survival based on the PKM2 IHC intensity score (High = 41, Low = 41). **e** Univariate and multivariate analyses of the OS of PDAC patients (*n* = 82 biological replicates). The dots on the forest plot represent the HRs of the Cox proportional hazards model, and the error bars are the two-sided 95% confidence intervals. *P < 0.05, **P < 0.01. Independent variables with *P* < 0.05 in the univariate analysis were included in the multivariate analysis. **f** Representative IHC staining image with anti-PKM2 and anti-PKM1 antibodies in patients with a high SUVmax (left) and a low SUVmax (right). The corresponding CT and PET images are shown on the right. Scale bar, 2 mm. Structures were manually annotated with Qupath: tumor (blue), TLS (green) and nerve (red). PKM1 is highly expressed in nerve areas only, whereas PKM2 is expressed ubiquitously. **g**, **h** Correlations between PKM2 intensity and the SUVmax (**g**) and between PKM2 intensity and the area of tumor-associated TLSs (**h**) from the PET/CT images of PDAC patients (*n* = 20 biological replicates). Statistical analysis: unpaired two-sided *t*-test (**c**); log-rank Mantel–Cox *t*est (**a**, **b**, **d**).

Given that the expression and subcellular localization of the PKM1 and PKM2 splice isoforms are highly context dependent and that their quantitatively different enzymatic activities contribute to distinct metabolic modes in tumors^10,19,26^, we first investigated the expression of PKM1 and PKM2 in primary human PDAC tumors. PKM2 mRNA and protein expression were upregulated in PDAC tumors (Fig. 1a left, c top), and both were associated with poor patient prognosis (Fig. 1a right, d). In contrast, both PKM1 mRNA and protein levels remained low (Fig. 1b left, c bottom) and were not correlated with prognosis (Fig. 1b right). Additionally, PKM2 was upregulated in PanIN lesions and neoplastic epithelium, compared with morphologically normal pancreas acinar cells, ductal cells, and regions of acinar-to-ductal metaplasia (ADM) (Fig. 1c; Supplementary Fig. S1a). Notably, morphologically normal pancreatic ductal cells had weak PKM2 expression, whereas acinar cells did not detectably express PKM2. PKM2 expression was upregulated during ADM progression (Supplementary Fig. S1a). Furthermore, PKM2 was highly expressed in fibroblasts, tertiary lymphoid structures (TLSs) (Supplementary Fig. S1b, green), and nerve cells (Supplementary Fig. S1b, red), whereas PKM1 was strongly expressed only in nerve cells (Supplementary Fig. S1b, red) and weakly expressed in vascular smooth muscle cells (Fig. 1c, black).

Owing to the undetectable levels of PKM1 in the neoplastic epithelium, we were unable to further evaluate its correlation with the prognosis of PDAC patients. However, we identified PKM2 as an independent prognostic factor for overall survival (OS) (hazard ratio (HR) = 2.1; 95% confidence interval (CI): 1.30–3.3; *P* = 0.003) and as an unfavorable factor for adjuvant therapy (HR = 0.48; 95% CI: 0.28–0.83; *P* = 0.008) (Fig. 1e). This correlation is consistent with a previous report that the modulation of alternative *PKM* splicing and elevated PKM2 expression promote gemcitabine resistance^27^.

^18^F-Fluorodeoxyglucose (^18^F-FDG) positron emission tomography/computed tomography (PET/CT) has been widely used to visualize the metabolic activity of tumors. We therefore examined PET/CT images from PDAC patients and the corresponding paraffin-embedded, immunohistochemistry (IHC) tumor tissue sections. PKM2 was extensively expressed in the neoplastic epithelium (blue), TLSs (green), and nerves (red), whereas PKM1 was strongly expressed only in nerves (red) (Fig. 1f). Additionally, the intensity of PKM2 expression was positively correlated with the maximum standardized uptake value (SUVmax) (Fig. 1g). By normalizing the PKM2-positive TLS area to the entire slide area, we found that the PKM2 intensity was negatively correlated with the TLS area in PDAC patient samples (Fig. 1h), indicating the potential benefit of modulating *PKM* splicing to improve the response to immunotherapy.

### Single-cell analysis reveals that tumor cells and activated T_regs_ rely on PKM2

**Fig. 2:**
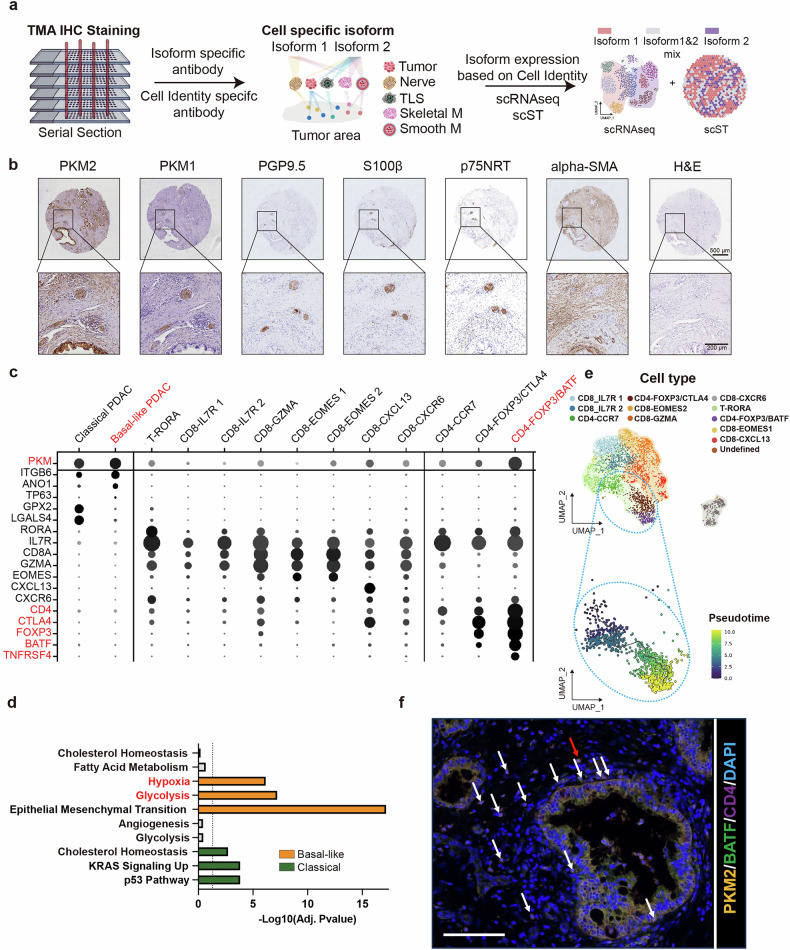
Broad screen for potential cells in the PDAC TME that may be targets of PKM-ASO. **a** Schematic and rationale of using scRNA-seq and scST to screen and show the localization of potential TME cell types that may be targeted by PKM-ASO. Well-annotated scRNA-seq data (*n* = 4 PDAC patients; biological replicates) from our previous studies were used^3,28^. The PKM1/PKM2 isoforms cannot be directly distinguished by scRNA-seq because of sparse coverage and because the only sequence differences between these isoforms map to exons 9 and 10, respectively. After removing nerve cells and Schwann cells (marked by *CDH19, PLP1*, and *SOX10*), which showed strong constitutive expression of PKM1 in our IHC data, *PKM* expression in the remaining cells in scRNA-seq and scST should be regarded as PKM2 rather than PKM1, providing the opportunity to identify potential cell types that can be targeted by PKM-ASO. **b** Representative IHC staining image with anti-PKM2, anti-PKM1, anti-PGP9.5, anti-S100β, anti-p75NRT, and anti-αSMA antibodies from serial sections of the TMA. Scale bar, 200 μm (bottom), 500 μm (top). PGP9.5 and S100β are nerve markers, p75NRT is a Schwann cell marker, and αSMA is a matrix marker. Images are representative of 83 PDAC samples with similar strong and constitutive PKM1 expression in nerves and ubiquitous PKM2 expression. **c** PKM2 expression across different cell types, annotated on the basis of the expression of marker genes. **d** GO analysis showing the different metabolic modes in basal-like and classical tumor cells. **e** UMAP plot showing 11 subclusters of T cells (top) and pseudotime analysis (bottom) of CD4-CCR7, CD4-FOXP3/CTLA-4, and CD4-FOXP3/BATF cells to show the dynamic differentiation and activation of T_regs_. CD4-CCR7 was set as the root for the pseudotime analysis. **f** Multi-IF staining of anti-PKM2, anti-BATF, anti-CD4, and DAPI. CD4-FOXP3/BATF cells surround the tumor area. Activated T_regs_ (arrows) co-express BATF, PKM2, and CD4 and surround PDAC cells. The image on the right is a 2.5-fold magnified view of the activated T_regs_ indicated by the red arrow. Scale bar, 0.1 mm. *P < 0.05, **P < 0.01, ***P < 0.001. The Benjamini‒Hochberg method was used to correct for multiple hypothesis testing (**d**).

To investigate the cell types that depend on PKM2 and that could be targeted to inhibit PDAC progression using our ASO, we reanalyzed a well-annotated single-cell RNA sequencing (scRNA-seq) dataset from patients with untreated, resectable, non-metastatic PDAC (*n* = 4)^28^. On the basis of the above PKM1 and PKM2 immunohistochemical staining results, we excluded minor cell types with strong expression of PKM1 only and focused on the remaining cell types that exhibited strong PKM2 expression (Fig. 2a, b). We investigated the unbiased most differentially expressed genes (DEGs) in addition to PKM2 expression (Fig. 2c). Uniform manifold approximation and projection (UMAP) analysis revealed that tumor cells formed two distinct subclusters that we annotated as basal-like (34.6%, 675/1952) and classical (65.4%, 1277/1952) PDAC cells, as previously described (Fig. 2c; Supplementary Fig. S2a)^29^. Compared with classical PDAC cells, basal-like PDAC cells exhibited higher expression of *PKM* (log_2_FC = 0.5, adj. *P* = 0.03) as well as other glycolysis hub genes, such as *LDHA* (log_2_FC = 0.63, adj. *P* = 2.85E-09), *ENO1* (log_2_FC = 1.03, adj. *P* = 4.63E−24) and *SLC2A3*, which encodes the glucose transporter GLUT3 (log_2_FC = 1.5, adj. *P* = 0.001). Basal-like PDAC cells were also enriched in the epithelial–mesenchymal transition (EMT) pathway, hypoxia, and glycolysis, whereas classical PDAC cells were not significantly enriched in glycolysis or angiogenesis (Fig. 2d). Similarly, cancer-associated fibroblast Cluster 2 (CAF2) (no significant difference in *PKM* expression), macrophage Cluster 3 (mφ3) (log_2_FC = 0.4, adj. *P* = 7.28E−88), and tumor-associated neutrophil Cluster 1 (TAN1) (log_2_FC = 1.5, adj. *P* = 1.02E−43) also exhibited highly enriched glycolysis (Supplementary Fig. S2a).

Notably, compared with naive CD4^+^ T cells (which express CD4/CCR7), terminally differentiated T_regs_ (which express *BATF*/*TNFRSF4*/*CTLA4*^30^) expressed much higher levels of *PKM* (Fig. 2c, e; Supplementary Fig. S2b). Activated T_regs_ are an immunosuppressive subset of CD4^+^ T cells that can promote tumor progression by impairing an effective antitumor immune response^30^. We performed splicing analysis of published RNA sequencing (RNA-seq) data^6^ for nT_convs_ (naive conventional T cells, CD45RA^+^CD25^–^CD4^+^, corresponding to naive CD4^+^ T cells) and eT_regs_ (highly suppressive effector T_regs_, CD45RA^–^CD25^high^FOXP3^high^CD4^+^, corresponding to activated T_regs_) to determine their PK isoform expression patterns. Mutually exclusive splicing analysis of *PKM* revealed that the average exon 9 inclusion values were 18% and 9% in nT_convs_ and eT_regs_, respectively (Supplementary Fig. S2c, d). Transcript counts revealed that both nT_convs_ and eT_regs_ preferentially expressed PKM2 rather than PKM1 (Supplementary Fig. S2e). This preference may be related to the need for immune cells to rapidly respond to external stimuli, as glycolysis promotes rapid ATP production and immune cell proliferation, supplies metabolic intermediates, and regulates cellular functions to support immune responses^31^. PKM2 expression remained elevated during T_reg_ differentiation, which is consistent with our scRNA-seq data analysis (Fig. 2c). Additionally, the expression of activated T_reg_ markers, such as *FOXP3*, *BATF*, *TNFRSF4*, and two immune checkpoint genes, *CTLA4* and *PD1*, was markedly elevated in eT_regs_ in this bulk RNA-seq dataset (Supplementary Fig. S2f).

Multiple immunofluorescence (IF) staining assays revealed that activated T_regs_ surrounded the tumor epithelium (Fig. 2f). In addition, our data suggested that tumor cells attracted activated T_regs_ via the receptor–ligand pairs C3-C3AR1, APP-CD74, LPAR2-ADGRE5, CD55-ADGRE5, CD47-SIRPG, and PPIA-BSG (Supplementary Fig. S3a). Interaction between complement C3 and C3AR1 was observed only between basal-like cells and activated T_regs_, but not between classical cells and activated cells, and was associated with poor prognosis in the TCGA-PAAD cohort (Supplementary Fig. S3,c). Treatment of BxPC-3 cells, a typical basal-like PDAC cell line, with a *PKM* splice-switching ASO, ASO1-TMO (Supplementary Table S2), induced significant splice switching, followed by slight downregulation of the expression of basal-like markers and C3 (Supplementary Fig. S3,b, d). Thus, our results demonstrate that tumor cells and the TME, especially basal-like PDAC cells and activated T_regs_, are potential targets of our PKM-ASO.

### Microarray-based spatial transcriptomics reveals that activated T_regs_ that surround basal-like tumor cells are highly glycolytic and have undergone EMT

**Fig. 3:**
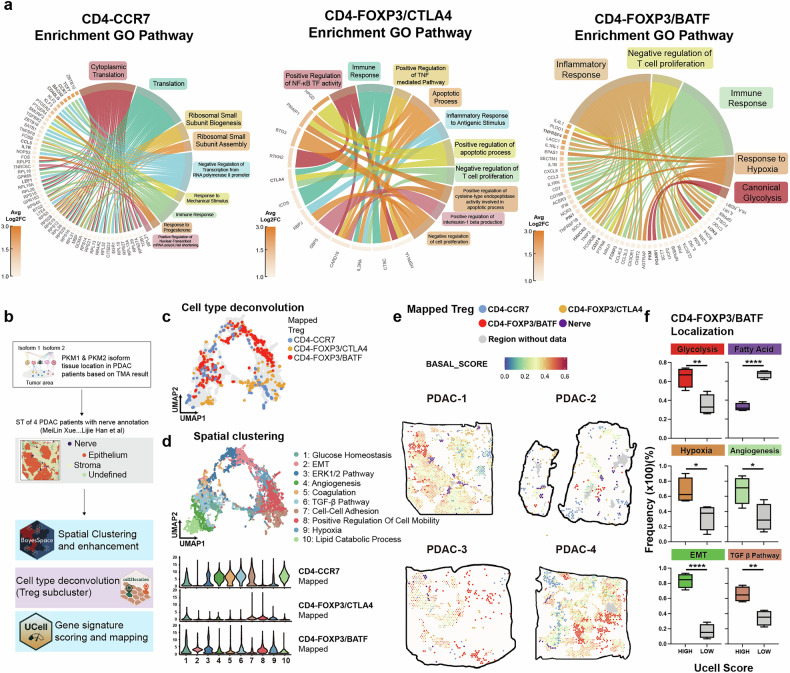
Microarray-based spatial transcriptomics reveals that basal-like tumor cells are surrounded by CD4-FOXP3/BATF cells. **a** GO analysis of the top 50 genes in each cell cluster. A *P* value < 0.05 was used for further visualization. Hypoxia and glycolysis pathways are enriched in CD4-FOXP3/BATF cells. **b** Schematic diagram of the scST analysis process. BayesSpace was used for spatial clustering, and the set of TOP50 genes in each cluster was input into EnrichR for GO analysis and visualization via a heatmap and UMAP. Cell2location was used for cell-type deconvolution on the basis of our scRNA-seq analysis. Then, CD4-CCR7, CD4-FOXP3/CTLA-4, and CD4-FOXP3/BATF cells were directly mapped onto the scST. We followed the previous annotation for the nerve, epithelial, stroma, and undefined regions in the scST^2^. Finally, UCell was used for signature scoring and visualization in UMAP as well as scST. Hallmark gene signatures were obtained from MSigDB. **c** Based on Cell2location, CD4-CCR7, CD4-FOXP3/CTLA-4 and CD4-FOXP3/BATF cells were mapped onto UMAP produced by BayesSpace. **d** GO analysis of each cluster shown in UMAP (top); violin plots show the frequency of CD4-CCR7, CD4-FOXP3/CTLA-4, and CD4-FOXP3/BATF cells in each GO cluster (bottom). **e** Spatial mapping of CD4-CCR7, CD4-FOXP3/CTLA-4, CD4-FOXP3/BATF, and nerve cells in each section (*n* = 4 biological replicates). First, UCell was used to calculate the BASAL-SCORE for epithelial cells, which were subsequently mapped onto sections to determine the spatial proximity between basal-like cells and T_regs_. The PDAC basal-like tumor signature was obtained from a previous work^29^. **f** Frequency of CD4-FOXP3/BATF cells localized in high/low signature score areas (*n* = 4 biological replicates). The signature was obtained from MSigDB, and UCell was used for evaluation. Statistical analysis: unpaired two-sided *t*-test (**f**); Benjamini‒Hochberg method to correct for multiple hypothesis testing (**a**).

We identified an intimate connection between basal-like PDAC cells and activated T_regs_ in the scRNA-seq data, but the localization of these two cell types in the immune microenvironment is unclear. To better understand the cross-talk between tumor cells and T_regs_ on the basis of their spatial distribution within tumors, we first investigated the functional differences among CD4-CCR7, CD4-FOXP3/CTLA-4, and CD4-FOXP3/BATF cells by performing Gene Ontology (GO) enrichment analysis using the scRNA-seq data. CD4-CCR7 cells were enriched in pathways related to the negative regulation of transcription, translation, and immune response, which is consistent with their immature phenotype and potential to differentiate (Fig. 3a, left). CD4-FOXP3/CTLA-4 cells were enriched in pathways associated with negative regulation of T-cell proliferation, inflammatory response to antigenic stimulus, and positive NF-κB transcription factor activity^4^, which reflected T_reg_ maturation after stimulation (Fig. 3a, middle). Finally, CD4-FOXP3/BATF cells were enriched in pathways related to the inflammatory response, negative regulation of the T-cell response, response to hypoxia, and glycolysis, which reflect the adaptation of activated T_regs_ to the hypoxic TME and their reliance on aerobic respiration to maintain their functions, such as migration^4^ and immunosuppression^5^ (Fig. 3a, right). Notably, CD4-FOXP3/BATF cells highly expressed *PKM* and multiple immune checkpoint genes, such as *TNFRSF4*, *HAVCR2*, and *CD274* (Fig. 3a, right).

We next characterized how T_regs_ at different stages interact with the neighboring TME using previous spatial transcriptomics annotation and the ST analysis pipeline (Fig. 3b). After clustering by BayesSpace (Supplementary Fig. S4a, b), Cell2location revealed that CD4-CCR7, CD4-FOXP3/CTLA-4, and CD4-FOXP3/BATF cells mapped to different regions within the tumor (Fig. 3c). Activated T_regs_ utilize glycolysis to migrate into inflamed tissues^4^. Activated T_regs_ (CD4-FOXP3/BATF) were linked to hypoxia, cell mobility, and glucose homeostasis (Fig. 3d). In contrast, compared with activated T_regs_, CD4-CCR7 cells were highly enriched in lipid catabolic processes, reflecting their different metabolism; whereas CD4-FOXP3/CTLA-4 cells appeared to be in an intermediate transition stage from CD4-CCR7 to CD4-FOXP3/BATF cells (Fig. 3d). After mapping nerve cells (which express PKM1), the basal-like score for each epithelial area, and T_regs_, we found that activated T_regs_ were most likely to surround epithelial areas with high basal-like scores, especially in Sections 1 and 4. In contrast, the localization of CD4-CCR7 and CD4-FOXP3/CTLA-4 cells was more random and generally in the epithelial areas with low basal-like scores (Fig. 3e).

To assess whether the function of T_regs_ was reflected in their localization, all spots were first scored for glycolysis, fatty acid, hypoxia, angiogenesis, EMT, and the TGF-β pathway signature, with a 50th percentile cutoff used to determine low and high regions (Supplementary Fig. S4d). CD4-FOXP3/BATF cells were more frequently present in regions with high scores for glycolysis, hypoxia, angiogenesis, EMT, and TGF-β and less frequently in fatty acid metabolism of high-scoring regions, which is highly consistent with the presumed mechanism of action of our PKM-ASO (Fig. 3f). In contrast, CD4-FOXP3/CTLA-4 cells were preferentially located in regions with low hypoxia and EMT. Neither CD4-CCR7 nor CD4-FOXP3/CTLA-4 cells showed a metabolic preference (Supplementary Fig. S4c). Thus, blocking the mutual attraction between basal-like PDAC cells and activated T_regs_ could be a potential therapeutic strategy.

### Basal-like PDAC cells are more sensitive to PKM-ASO than classical PDAC cells

**Fig. 4:**
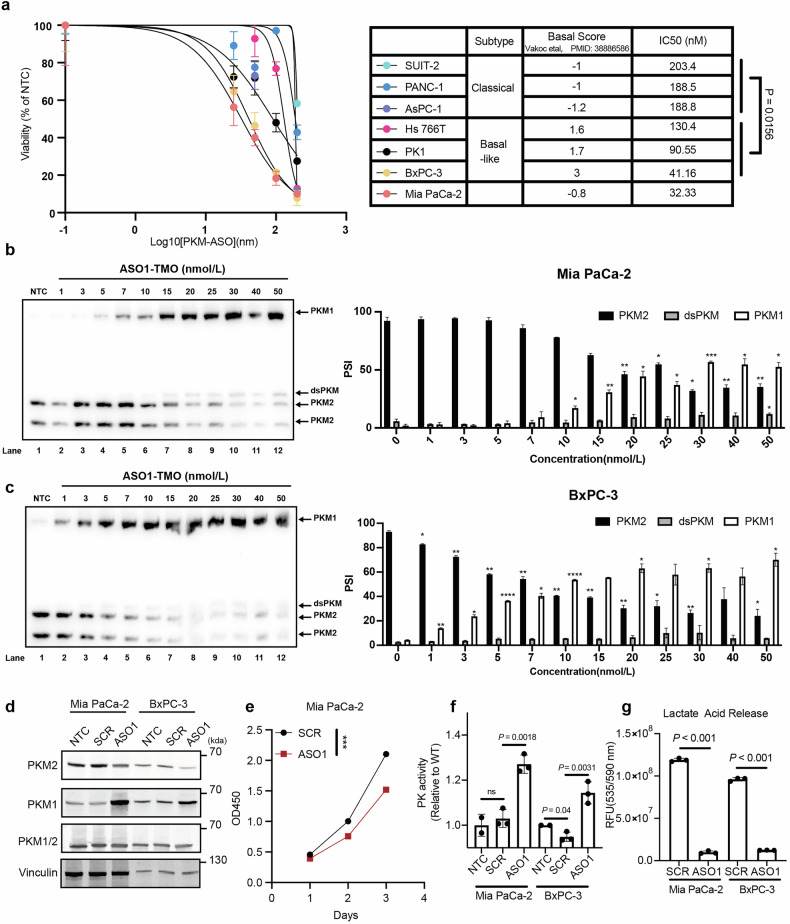
Transfection of ASO1-TMO induces *PKM* splice switching in PDAC cells. **a** IC_50_ of ASO1-TMO. Three classical PDAC cell lines (SUIT-2, PANC-1, and AsPC-1), two basal-like PDAC cell lines (Hs 766 T and BxPC-3), and the MIA PaCa-2 cell line were used for IC_50_ determination. Cells were transfected with Lipofectamine and varying concentrations (0, 25, 50, 100, and 200 nM) of ASO1-TMO on Day 0, 10^3^ cells were plated on a 96-well plate on Day 1, and cell viability was monitored by a colorimetric assay on Day 3. **b** Radioactive RT-PCR showing the extent of *PKM* splice switching after MIA PaCa-2 cells were transfected with the indicated concentrations of ASO1-TMO for 2 days (left). ImageJ was used to quantify the expression of the PKM2, PKM1, and PKMds isoforms (right). **c** Radioactive RT-PCR results showing the extent of *PKM* splice switching after BxPC-3 cells were transfected with various concentrations of ASO1-TMO as described in **b** for 2 days (left). Isoform quantification in **b** (right). **d** Western blot analysis showing the extent of isoform switching after MIA PaCa-2 or BxPC-3 cells were transfected with 50 nM ASO for 3 days. NTC, no-treatment control. **e** Viability of MIA PaCa-2 cells at each time point. Cells were transfected with 50 nM ASO on Day 0, 0.5 × 10^3^ cells were plated on a 96-well plate on Day 1, and the OD450 was monitored daily by a colorimetric assay. **f** PK activity was measured on Day 3 after transfection with 50 nM ASO. **g** Lactic acid levels were measured on Day 3 after transfection with 50 nM ASO. Statistical analysis: unpaired two-sided *t* test (**a**, **b**, **c**, **f**, **g**); two-way ANOVA (**e**).

The metabolic differences we observed between basal-like and classical PDAC cells suggested that these cells might respond differently to PKM-ASO (Fig. 2d). Although the expression of PKM2, PKM1, and the PKM2/PKM1 ratio did not differ between basal-like and classical cell lines, we detected differences in the expression of splicing regulators between these two subtypes (Supplementary Fig. S5a–e). ASO1-TMO effectively inhibited the proliferation of basal-like cell lines but had less of an effect on the proliferation of classical cell lines (Fig. 4a; Supplementary Fig. S5,d). To investigate the extent of splice switching in each subtype of PDAC cells, we transfected representative PDAC cell lines with varying concentrations of ASO1-TMO and analyzed the extracted RNA by radioactive RT-PCR. As expected, ASO1-TMO simultaneously increased the expression of PKM1 mRNA, decreased that of PKM2 mRNA, and increased that of PKMds mRNA, an isoform that is subject to nonsense-mediated mRNA decay (NMD), because of the presence of a premature termination codon in exon 11 when both exons 9 and 10 are skipped^32^ (Fig. 4b, c). Notably, splice switching occurred at a lower ASO concentration in BxPC-3 cells (EC_50_ = 8.55 nmol/L, basal score = 3) than in MIA PaCa-2 cells (EC_50_ = 23.44 nmol/L, basal score = –0.8). Consistent with the RNA data, *PKM* splice switching led to an increase in PKM1 and a decrease in PKM2 protein levels without changing total PKM expression in both MIA PaCa-2 and BxPC-3 cells (Fig. 4d). Collectively, these results confirm that our ASO1-TMO induces *PKM* splice switching and suggest that, compared with classical PDAC cells, basal-like PDAC cells are more sensitive to ASO1-TMO.

To further validate the antitumor efficacy of ASO1-TMO, MIA PaCa-2 and BxPC-3 cells were transfected with 50 nmol/L ASO and they showed significantly reduced proliferation compared with cells transfected with the scrambled sequence control, SCR-TMO (Fig. 4e; Supplementary Fig. S5,f). PKM1 is a constitutively active tetramer, whereas PKM2 is allosterically regulated and exists as either a catalytically active tetramer or a low PK activity dimer and monomer^10^ (Supplementary Fig. S5,g). ASO1-TMO significantly reduced the expression of PKM2 monomers/dimers, which are essential for aerobic glycolysis in tumors. Considering that ASO1-TMO induced splice switching from PKM2 to PKM1 and therefore depleted the low-activity PKM2 monomer/dimer, we expected that PK activity would increase and that glycolysis would decrease following ASO1-TMO treatment. Consistent with our previous work^22^, total PK activity significantly increased after ASO1-TMO treatment in both MIA PaCa-2 and BxPC-3 cells (Fig. 4f), and the level of the terminal glycolysis metabolic product lactate significantly decreased by approximately 12-fold and 8-fold in MIA PaCa-2 and BxPC-3 cells, respectively (Fig. 4g). Interestingly, treatment with si-PKM2 (Supplementary Fig. S5,h) decreased BxPC-3 cell proliferation but only had a limited effect on MIA PaCa-2 cells (Supplementary Fig. S5,i). Additionally, overexpression of PKM1 (Supplementary Fig. S5j) did not affect the proliferation of either cell line (Supplementary Fig. S5k). We conclude that the inhibition of cell proliferation caused by ASO1-TMO was due to the downregulation of PKM2 rather than the upregulation of PKM1. Our observations above revealed that the proliferation of MIA PaCa-2 cells decreased only slightly upon si-PKM2 treatment (with one of two siRNAs; Supplementary Fig. S5i) but that these cells were sensitive to ASO1-TMO treatment (Fig. 4a, e), which might reflect the fact that PKM1 can also promote PKM2 tetramer formation^19^ (Supplementary Fig. S5g).

### PKM-ASO inhibits MIA PaCa-2 cell growth in an immunodeficient mouse model

**Fig. 5:**
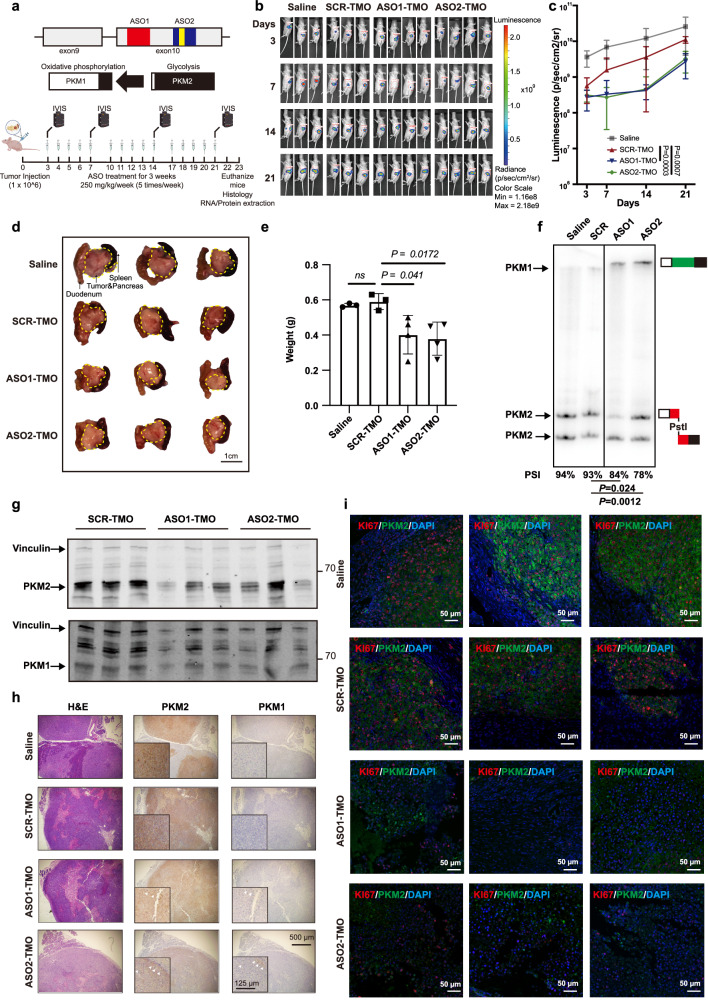
ASO1-TMO and ASO2-TMO inhibit the growth of MIA PaCa-2 orthotopic xenografts. **a** Schematic diagram of the animal experiment. ASO1-TMO and ASO2-TMO target the human *PKM* exon 10 regions (red and blue, respectively) that we previously targeted with MOE ASOs^60^. ASO2-TMO targets an SRSF3 binding site^60^ that is conserved between humans and mice (yellow). **b** Whole-animal live imaging of luciferase-expressing MIA PaCa-2 cells transplanted into the pancreas. Luminescence images of the transplanted mice on the indicated days are shown with a color scale in photons/sec/cm^2^/steradian. A total of 14 mice were randomized to each treatment group. **c** Quantification of the luciferase signal in (**b**). Statistical analysis: two-way ANOVA. **d** Images of representative tumors from animals treated as indicated on Day 23. The duodenum, pancreas, tumor, and spleen are shown. Dashed lines delineate the tumor. Scale bar, 1 cm. **e** Quantification of tumor weight on Day 23 (after the duodenum, normal pancreas, and spleen were removed). **f** ASO1-TMO and ASO2-TMO induce *PKM* splice switching in pancreatic tumors, as determined by radioactive RT-PCR of RNA from pancreatic tumor samples from tumor-bearing mice on Day 23. **g** Western blot analysis of PKM1, PKM2, and Vinculin in orthotopic tumor samples from mice treated with SCR-TMO, ASO1-TMO, or ASO2-TMO. **h** Representative H&E and IHC images of a tumor, showing the expression of PKM1 and PKM2. **i** Representative IF staining images of tumor sections from mice treated with saline, SCR-TMO, ASO1-TMO, or ASO2-TMO showing the expression of PKM2 (green), the proliferation marker Ki67 (red), and nuclei stained with 4′,6-diamidino-2-phenylindole (DAPI; blue). Scale bar, 50 µm. Statistical analysis: unpaired two-sided *t*-test (**e**, **f**); two-way ANOVA (**c**).

Before administering the ASO in vivo, we first evaluated whether ASO1-TMO delivered by free uptake (gymnosis) could induce *PKM* splice switching in the above cell lines. Consistent with the results of the above transfection assays, ASO1-TMO had a minor but significant splice-switching effect at approximately 20 µmol/L in both BxPC-3 and MIA PaCa-2 cells (Supplementary Fig. S6a, b). At this same ASO concentration, we also observed significantly slower proliferation and reduced G2/M cell cycle arrest in MIA PaCa-2 and BxPC-3 cells (Supplementary Fig. S6c, d) along with increased PK activity (Supplementary Fig. S6e) and decreased lactate production (Supplementary Fig. S6f). We then explored the in vivo efficacy of unformulated ASO1-TMO in an orthotopic xenograft model (Fig. 5a). To rule out potential off-target effects in vivo, we also used a second splice-switching ASO, ASO2-TMO (Supplementary Table S2), with a different *PKM* target sequence^22^, which showed efficacy comparable to that of ASO1-TMO (Fig. 5a; Supplementary Fig. S6g).

We used MIA PaCa-2 cells for the in vivo orthotopic xenograft experiments, as ASO treatment markedly inhibited their proliferation in vitro. After surgery, the tumors were allowed to engraft for 3 days prior to ASO treatment. The saline control group showed active tumor growth throughout the experiment (Fig. 5b). In contrast to the strong tumor inhibition induced by PKM-ASO treatment that was previously reported in two HCC mouse models^22^, the proliferation of xenografted PDAC tumors was relatively slow during the first two weeks but increased rapidly during the third week of PKM-ASO treatment (Fig. 5b, c). Nevertheless, the luciferase signal was significantly weaker in both ASO1-TMO-treated and ASO2-TMO-treated mice than in ASO-SCR-treated or saline-treated mice at the end of the experiment (Day 21) (Fig. 5c). PKM-ASO treatment was not associated with changes in body weight in this tumor model (Supplementary Fig. S7a). Additionally, fewer tumors were isolated from the pancreas of ASO1-TMO-treated and ASO2-TMO-treated mice on Day 23 than from ASO-SCR-treated mice (Fig. 5d, e).

Using radioactive RT-PCR, we detected significant splice switching in PDAC tumors following ASO treatment (Fig. 5f; Supplementary Fig. S7b). We also analyzed PKM2 and PKM1 protein expression, but detected only slight downregulation of PKM2 expression and did not consistently observe isoform switching after ASO treatment (Fig. 5g; Supplementary Fig. S7c). Considering the rapid tumor growth in the third week, isoform switching might be masked by negative selection or the development of treatment resistance (Fig. 5c).

We next performed IHC using anti-PKM2-specific and anti-PKM1-specific antibodies to evaluate metabolic heterogeneity. The PKM2 signal was slightly weaker in tumors treated with PKM-ASO, with a slight increase in the PKM1 signal in a minority of cells (Fig. 5h, arrow). To further characterize the localization of PKM-ASO and its effect, we first verified by IF that the ASO could be effectively delivered to PDAC tumors in vivo. As expected, we detected the ASO in PDAC tumor cells and a decrease in PKM2 expression, as well as a decrease in the nuclear area, indicating that these cells underwent necrosis or apoptosis (Supplementary Fig. S7e). We then focused on the necrotic areas on the basis of H&E staining. PKM-ASO significantly decreased the intensity of PKM2 and Ki67 staining (a proliferation marker) but did not significantly increase the intensity of PKM1 staining (Fig. 5i; Supplementary Fig. S7f, g). We conclude that treatment with PKM-ASO inhibits PDAC tumor growth in this xenograft model.

To evaluate the potential off-target and side effects of PKM-ASO, we analyzed kidney, liver, duodenum, and muscle tissues. We chose these tissues for the following reasons: first, ASOs generally accumulate in the kidney and liver, which are major organs affected by off-target toxicity^24^; second, muscle tissue expresses PKM1 rather than PKM2, so any histological changes there would be due to off-target effects; and third, the duodenum was reported to express PKM2^10^, making it relevant to evaluate histological changes and splice switching for assessing potential side effects (Supplementary Fig. S7h). A comprehensive histopathological assessment revealed no significant changes or toxic effects of PKM-ASO or ASO-SCR in the kidney (Supplementary Fig. S7h, k), duodenum, liver, or muscle (Supplementary Fig. S7h). PKM1 and PKM2 expression did not significantly change in the duodenum (Supplementary Fig. S7i). Furthermore, there was no significant splice switching observed in the kidney or duodenum (Supplementary Fig. S7j, l, m). Collectively, these results position PKM-ASO as an effective *PKM* splicing modulator with no signs of toxicity as a single agent in preclinical models of PDAC.

### Synergy between PKM-ASO and anti-CTLA-4 treatment in an immunocompetent PDAC mouse model

Considering the limited treatment efficacy of single anti-cancer agents, we evaluated potential synergistic treatments on the basis of the above PET/CT (Fig. 1f), scRNA-seq (Fig. 2c), and scST (Fig. 2e) data. Because PKM-ASO is also taken up by non-tumor cells, e.g., in our previous study of HCC^22^, and because inhibition of glycolysis can sensitize tumors to immunotherapy^7,8^, it was logical to consider the immune microenvironment in PDAC. We tested three potent murine *Pkm* ASOs on the basis of our previous screen^22^ with locked nucleic acid (LNA) or 2′-O-methoxyethyl (MOE) modifications along with two thiomorpholino (TMO)-modified ASOs whose target sequences overlap potential splicing factor binding motifs (Supplementary Fig. S8a and Table S2). We identified MOE16 as the most potent of these ASOs, which induced significant *Pkm* splice switching in both transfection and free uptake experiments with murine KPC1412 tumor cells (Supplementary Fig. S8b, c). Moreover, MOE16 strongly inhibited KPC1412 tumor cell growth in vitro in both the transfection and free uptake experiments (Supplementary Fig. S8d, e). The target site for MOE16 in *Pkm* partially overlaps the corresponding binding site for ASO2-TMO in the *PKM* ortholog, with only one base difference between the *H. sapiens* and *M. musculus* sequences. We also designed two MOE/PS-modified ASO controls, neither of which elicited splice switching compared with the MOE16-treated group nor had any obvious effect on KPC1412 cell viability compared with the NTC group (Supplementary Fig. S8f). We selected mSCR2 as a scrambled sequence control ASO for further in vivo study.

**Fig. 6:**
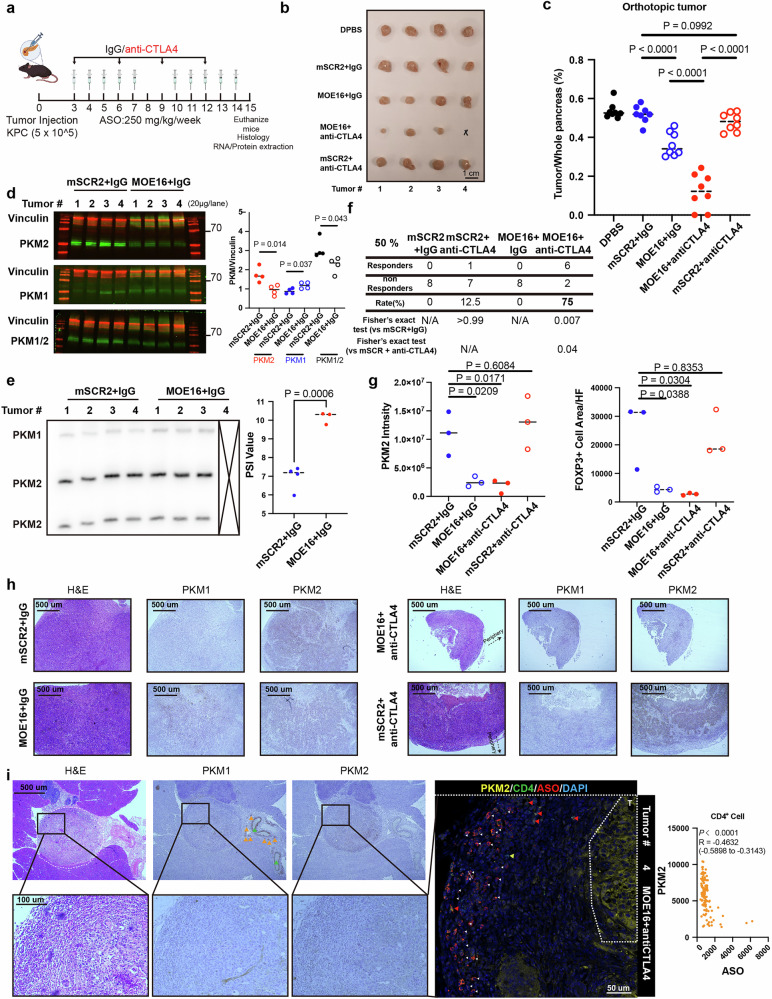
ASO-based *Pkm* splice switching increases the response to anti-CTLA-4 therapy in PDAC models. **a** Schematic diagram of the experiment in C57/BL-6J mice. MOE16 (see Supplementary Fig. S8a) targets a conserved SRSF3 binding site and was delivered i.p. with a weekly schedule of 5 consecutive injections followed by 2 days of rest for 2 weeks. One hundred micrograms of mouse IgG2b control antibody or anti-CTLA-4 antibody was delivered i.p. every 3 days. **b** Images of representative tumors on Day 15; scale bar, 1 cm. **c** Tumor weight/weight of the whole pancreas on Day 15 (*n* = 8 biological replicates per group). **d** *PKM* splice switching reflected at the protein level in the mSCR2 + IgG and MOE16 + IgG groups (red, Vinculin, 124 kDa; green, PKM1, PKM2, total PKM, 60 kDa). ImageJ was used to quantify the PKM1, PKM2, and total PKM protein levels. **e** Radioactive RT-PCR of *Pkm* splice switching in tumors 15 days after i.p. injection of ASO. Tumor #4 in Row 3 of Panel b was not included because of low RNA quality. ImageJ was used to quantify the expression of the Pkm1 and Pkm2 isoforms. **f** Response rates measured from two independent experiments. Response was defined as a > 50% reduction in tumor weight compared with that of control tumors (mSCR2 + IgG). N/A, not applicable. **g** PKM2 intensity (left, based on **h**) and FOXP3^+^ cell area (right, based on Supplementary Fig. S9f) in each treatment group. **h** H&E staining and IHC results showing *Pkm* splice switching 15 days after i.p. injection of MOE16 or mSCR2 ASO and the anti-CTLA-4 or IgG antibody. The black arrow shows the peripheral area of the tumor. Scale bars, 500 μm (inset). **i** H&E staining, IHC, and IF staining results showing a pancreatic tumor in remission (tumor #4 in the 4^th^ row of Panel b, following anti-CTLA4 and MOE16 ASO treatment). The white dashed line indicates tumor remnants. Scale bars, 250 and 100 μm (inset). Orange triangle: nerve. Green triangle: vessel. White triangle: ASO^+^CD4^+^PKM2^–^ cells. Red triangle: ASO^–^CD4^+^PKM2^+^ cells. Yellow triangle: ASO^–^CD4^+^PKM2^–^ cells. Zen was used to quantify the intensity of PKM2 and ASO in CD4^+^ T cells, and Pearson correlation analysis was performed. IHC experiments in **h** (including treatments with scrambled sequence ASO and IgG) and 6i were performed in parallel. Statistical analysis: unpaired two-sided *t* test (**c**, **d**, **e**, **g**); Pearson correlation analysis (**i**).

To determine whether inducing *Pkm* splice switching can synergize with anti-CTLA-4 therapy and sensitize PDAC cells to ICB, we treated established orthotopic allograft PDAC tumors with an anti-CTLA-4 antibody alone or in combination with MOE16 (Fig. 6a). Consistent with the results of previous pre-clinical experiments^33^ and those of a clinical trial^34^, the tumors from the control animals did not respond to anti-CTLA-4 treatment (Fig. 6b, c, f; Supplementary Fig. S9a). The tumors from animals treated with MOE16 alone were slightly smaller, which is consistent with our results in the immunodeficient mouse model (Fig. 6b, c; Supplementary Fig. S9a). Compared with MOE16 + IgG and mSCR2 + IgG, combined treatment with MOE16 plus anti-CTLA-4 had a synergistic effect, significantly decreasing tumor weight (Fig. 6b, c; Supplementary Fig. S9a). Notably, 2/8 tumors were undetectable by visual inspection. Moreover, this combination therapy increased the response rate to anti-CTLA-4 treatment (anti-CTLA-4 + mSCR2 vs IgG + mSCR2 = 12.5%, MOE16 + anti-CTLA-4 vs MOE16 + IgG = 75%) (Fig. 6f) without obvious toxicity (Supplementary Fig. S9b, c). Surprisingly, MOE16 treatment resulted in a slight increase in body weight (Supplementary Fig. S9b). Systemic delivery of MOE16 induced splice switching in PDAC tumors, the duodenum and kidney and the corresponding protein isoform changes in PDAC tumors (Fig. 6d, e, h; Supplementary Fig. S9,d, e). The tumors exhibited decreased infiltration of FOXP3^+^ T_regs_, decreased *Pkm2* mRNA expression, and increased *Pkm1* mRNA expression (Fig. 6g, h; Supplementary Fig. S9f, g). Anti-CTLA-4 monotherapy did not significantly affect T_reg_ infiltration in PDAC tumors, but Pkm-ASO monotherapy or combination therapy effectively reduced FOXP3^+^ T_reg_ infiltration (Fig. 6g). CD4^+^ T cells that accumulated ASOs in the cytosol had decreased PKM2 expression (white triangle), whereas CD4^+^ T cells without ASO accumulation in the cytosol had high PKM2 expression (red triangle) (Fig. 6i; Supplementary Fig. S9h, i).

## Discussion

**Fig. 7:**
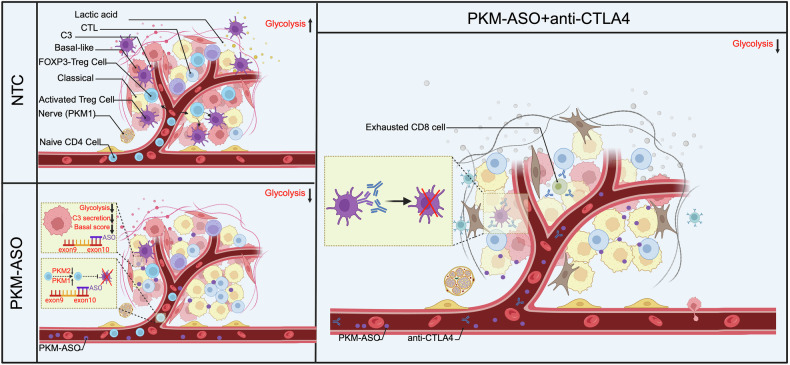
Systemic delivery of PKM-ASO reprograms the TME and intratumoral metabolism. Although PKM-ASO monotherapy has limited effects in an immunodeficient mouse model, synergy between PKM-ASO and ICB via an anti-CTLA-4 antibody inhibits PDAC progression by decreasing T_reg_ infiltration, thereby increasing the response rate of immune “cold” tumors to ICB.

The observation that the systemic delivery of PKM-ASO leads to its broad distribution in the TME motivated us to explore specific cell types dependent on PKM2 and to investigate a potential combination therapy^22^. However, neither droplet-based nor plate-based scRNA-seq methods can achieve sufficient sequencing depth to provide isoform information, thus limiting their utility in investigating the TME^35^. Fortunately, in our large PDAC tumor microarray (TMA), PKM1 and PKM2 exhibited distinct tissue-specific expression patterns. Combining this information with previously reported PDAC nerve and stromal TMA immunohistochemical staining data, along with scRNA-seq and scST data, we demonstrated that PKM1 is expressed exclusively in nerve and smooth muscle, whereas PKM2 is expressed nearly ubiquitously. Therefore, after excluding the cell types that expressed only PKM1, the expression of *PKM* in the remaining cell types according to the scRNA-seq and scST data corresponded to that of PKM2 rather than to that of PKM1, thus providing the opportunity to screen potential cell types that can be effectively targeted by PKM-ASO. On the basis of this assumption, we identified basal-like PDAC cells and their surrounding activated T_regs_ as cell types that rely on PKM2 to sustain glycolysis (Fig. 7).

Despite the fact that PKM1 and PKM2 differ by only a few amino acids, they play very different roles in tumors, which may depend on the tumor type. Unlike in HCC cells, in which PKM-ASO inhibits tumor growth through the combined effect of PKM1 upregulation and PKM2 downregulation^22^, we determined that decreased PKM2 expression rather than increased PKM1 expression is important for PDAC cells by using isoform-specific PKM2 siRNA, doxycycline-inducible PKM1 expression, and a TMA. Nevertheless, experiments that modulate only one isoform cannot fully recapitulate the integrated biological consequences of ASO treatment, which simultaneously and reciprocally alters both isoforms. A previous study revealed that deletion of the PKM2-specific exon 10 does not affect overall survival in a KP^–/–^C PDAC mouse model^36^. The discrepancy between survival outcomes in PDAC patients and mouse models may be attributable to selection pressure. As indicated by our scRNA-seq data, although the expression levels of PKM2 differed only slightly across tumor subtypes, the metabolic heterogeneity between subtypes influenced their response to PKM-ASO. A previous scRNA-seq dataset from KPC mice revealed that tumor cells could be classified into three clusters: a classical epithelial cell state, a basal epithelial cell state, and a mesenchymal cell state^37^. The mesenchymal cell state does not exhibit obvious glycolysis or OXPHOS, but it is clinically correlated with poor overall survival in PDAC patients^23^. Although PKM2 knockdown indeed inhibited PDAC cell proliferation in our study, selection pressure may have allowed a cluster of malignant cells without a clear metabolic mode to persist. This may partially explain the contradictory results described for the role of PKM2 in breast cancer^38^, pancreatic cancer^36^, and HCC^39^. Additionally, selection pressure could explain the modest changes in *PKM* splice switching and PKM2 protein levels observed in tumors after ASO treatment. Notably, the PDAC patient-derived scRNA-seq data are from patients with untreated, resectable, non-metastatic pancreatic tumors^3^, whereas KP^–/–^C mice have more aggressive disease^40^. Thus, the proportion of malignant basal-like and mesenchymal subtypes is likely greater in KP^–/–^C mice than in patients, suggesting the need to consider disease stage and tumor selection pressure before treating patients with PKM-ASO.

The scRNA-seq and scST analyses provide a new perspective for PDAC treatment. Unlike the inconsistent role of PKM in different cancers, MYC controls metabolic reprogramming and promotes PKM2 expression during T-cell activation^41,42^. Knockout of PKM2 exon 10 in CD8^+^ T cells decreases glycolytic flux and increases the activity of the pentose phosphate pathway, resulting in synergistic durable antitumor immunity and ICB in murine NSCLC and melanoma models^43^. Moreover, pharmacological activation of PKM2 inhibits CD4^+^ T-cell differentiation into T_regs_ under polarizing conditions (TGF-β treatment)^44^. Our data demonstrate significant increases in PKM2 expression and slight but significant *PKM* splice switching during T_reg_ differentiation. Activated T_regs_ were also enriched in the TGF-β pathway and were localized mainly around the tumor area, indicating an intimate relationship with basal-like PDAC cells. Consistent with the findings of a previous report^4^, compared with naive CD4^+^ T cells, activated T_regs_ exhibit increased glycolysis and migration ability. Importantly, after ASO treatment, T_reg_ infiltration into the TME decreased, providing a theoretical basis for its combination with ICB. Although PKM-ASO may have a limited effect, due to variations in genetic background or tumor subtype, limiting T_reg_ infiltration may be beneficial in the context of tumors with immunosuppressive TMEs, such as PDAC, diffuse midline glioma (DMG), and ICB-resistant melanoma.

In this study, we focused on T_regs_ and basal-like PDAC cells, given their intimate spatial relationship and reliance on PKM2 in the TME. However, we also detected *PKM* expression in other immune cells, such as exhausted CD8^+^ T cells, tumor-associated neutrophils, and macrophages, in our scRNA-seq data. Investigating the consequences of changes in *PKM* expression in these and other immune cell types, such as via PKM-ASO-mediated metabolic reprogramming or via PKM2-elicited transcriptional changes, are important future direction.

In addition to its canonical functions in metabolism, the roles of PKM2 in immune regulation and tumor biology are being increasingly recognized^45^. Nuclear PKM2 can act as a transcriptional coactivator of HIF-1α, STAT3, and β-catenin, thereby influencing cytokine production, T-cell differentiation, and tumor-associated immune responses independent of glycolysis^44,46^. These non-metabolic activities may contribute to the immunosuppressive phenotype we observed in T_regs_ and their crosstalk with basal-like PDAC cells. Given these emerging findings, determining the metabolic and non-metabolic functions of PKM2 in T_reg_ biology and PDAC progression will be an important future direction.

Our study has several limitations. First, we used two acute PDAC models in which tumors developed within one month. Although rapid tumor formation accelerated this study, it may have limited the efficacy of the therapy. Because the majority of PDAC patients suffer from chronic pancreatitis during disease progression, spontaneous pancreatic cancer models, such as KPC (LSL-Kras^G12D/+^; LSL-Trp53^R172H/+^; Pdx1-Cre) mice that mimic PDAC TME progression should be considered for use in future work. Second, it will be of interest to test patient-derived xenograft (PDX) or patient-derived organoid (PDO) models to maintain intratumor heterogeneity and to compare PDAC tumors with different TMEs rather than using immortal cell lines that have lost heterogeneity, although such models lack an intact immune microenvironment. Third, we used repeated doses of ASO in our in vivo experiments; although this did not result in obvious toxicity, the minimum effective and maximum tolerated doses should be determined in the future. Fourth, Smart-seq of relevant cells directly after PKM-ASO treatment should be considered; our study provided a preliminary classification of the TME, and further Smart-seq analysis may provide sufficient resolution for detailed characterization of PKM-ASO-induced subtype transitions and of cell differentiation as a function of PKM isoform expression. Fifth, humanized mouse models or human *PKM* knock-in mouse models should be considered for use to allow preclinical testing of human-specific ASOs.

In summary, the systemic delivery of PKM-ASO can reprogram the TME and intratumoral metabolism. Although PKM-ASO monotherapy had a limited effect in an immunodeficient mouse model, synergy between PKM-ASO and anti-CTLA-4 ICB significantly inhibited PDAC progression by decreasing T_reg_ infiltration, thereby increasing the response rate of immune “cold” tumors to ICB.

## Materials and methods

### Patients

Primary PDAC tissues and tumor-adjacent tissues (> 3 cm from the tumor edge) were surgically resected from patients at Ruijin Hospital (Shanghai, China) between January 2016 and July 2021. This study was approved by the Ruijin Hospital Ethics Committee (reference number: 2013-70). All patients provided informed consent. Patients who met the following criteria were enrolled in the study: (1) the histopathological diagnosis was PDAC; (2) they had not received any prior anti-cancer treatments; (3) they had no history of other malignancies; and (4) follow-up was completed within the scheduled time frame. A total of 83 pairs of normal pancreata and PDAC tissues were obtained from surgical specimens for tissue microarrays, including those from 48 males and 34 females with median ages of 62 and 67 years, respectively. Clinical information included gender, age, pathologic diagnosis, degree of differentiation, American Joint Committee on Cancer (AJCC) stage, date of surgical resection, status of cancer recurrence, survival status, chemotherapy information after surgery, and PET/CT images.

### H&E, IHC, and IF

H&E staining, IHC, and IF staining of PDAC tissues from archived, formalin-fixed, paraffin-embedded (FFPE) tumors were performed according to standard protocols. For IHC, the slides were incubated with primary antibodies against PKM2 (1:200; Cell Signaling Technology, rabbit monoclonal, 4053S), PKM1 (1:200; Cell Signaling Technology, rabbit monoclonal, 7067S), CD3ε (1:100; Cell Signaling Technology, rabbit monoclonal, 99940 T), FOXP3 (1:100, Abcam, rabbit monoclonal, ab215206), PGP9.5 (1:500, Servicebio, rabbit polyclonal, GB11159), S100β (1:150, Servicebio, GB11359), p75NRT (1:100, Abcam, rabbit monoclonal, ab52987), α-SMA (1:200, Abcam, rabbit polyclonal, ab5694), Ki67 (1:50; BD Biosciences, mouse monoclonal, B56), ModDetect™ phosphorothioate detection reagent (1:1000, Rockland, mouse polyclonal, 200-301-MV1S), BATF (1:500, Abcam, rabbit polyclonal, ab244528), and CD4 (1:400, Proteintech, mouse monoclonal, 67786-1-Ig). For IHC, the signal was visualized with a horseradish peroxidase-labeled anti-rabbit polyclonal antibody (1:200; Agilent, P0448) and 3,3’-diaminobenzidine (Agilent, K346711). Slides were counterstained with hematoxylin (Epredia, 7211), and images were captured with a Zeiss Observer microscope. For IF, the signal was visualized with fluoro-conjugated secondary antibodies (Thermo Fisher Scientific, goat anti-mouse immunoglobulin G (IgG) (H + L) Highly Cross-Adsorbed, Alexa Fluor Plus 555, A32727 or goat anti-rabbit IgG (H + L) Highly Cross-Adsorbed, Alexa Fluor Plus 488, A32731). Before being incubated with the mouse-derived anti-ASO antibody, mouse tissues were treated with an M.O.M. kit (Vector Labs, BMK-2202) to reduce endogenous mouse IgG staining, and the ASO was visualized with a streptavidin and Alexa Fluor 555 Conjugate (Thermo Fisher Scientific, S21381). Slides were counterstained with 0.1 μg/mL DAPI (Thermo Fisher Scientific; USA), and images were captured on a Zeiss LSM710 confocal laser scanning microscope.

### scRNA-seq and statistical analysis

The scRNA-seq data were analyzed as previously described^47^. The adapter sequence was filtered using Fastp with the default parameters, after which low-quality reads were removed^48^. To identify the cell barcode whitelist, UMI tools were used^49^. Mapping to the human genome (Ensembl v91) was performed using STAR mapping^50^ with customized parameters from the UMI-tools standard pipeline, and the UMI counts of each sample were obtained. Cells with more than 200 expressed genes and mitochondrial UMI rates less than 20% passed the cell quality filter, and mitochondrial genes were removed. The Seurat package (v5.1.0, https://satijalab.org/seurat/) was used for cell normalization and regression on the basis of the expression table, according to the UMI counts of each sample and the percentage of mitochondria, to obtain scaled data.

Utilizing the graph-based cluster method (resolution = 0.5), we acquired the unsupervised cell cluster result on the basis of the principal component analysis (PCA) of the top 30 principal components, and we calculated the marker genes using the FindAllMarkers function with the Wilcoxon rank-sum test algorithm using the following criteria: (1) logFC > 0.25; (2) *P* value < 0.05; and (3) min.pct > 0.1.

To identify cell types in detail, clusters of the same cell type were selected for re-UMAP analysis, and graph-based clustering, marker analysis, and Enrichr^51^ or DAVID (https://davidbioinformatics.nih.gov/) were further performed for each cluster.

### Pseudotime trajectory analysis

Pseudotime analysis was performed to determine the differentiation of T_regs_ using^52^ (v 0.2.2.0; https://github.com/cole-trapnell-lab/monocle3). The expression data were UMAP-embedded using the Monocle function “reduce_dimension” with default parameters. The trajectory graph was inferred with the function “learn_graph”, with the minimal branch length set to 15 and close_loop = FALSE. CD4 trajectories were rooted in the clusters CD4-CCR7.

### Cell–cell communication analysis

CellPhoneDB analysis (https://github.com/Teichlab/cellphonedb) was applied to analyze cell‒cell communication between cell types of interest^53^ on the basis of the normalized expression matrix from Seurat. The mean expression of each receptor–ligand pair was calculated as the mean of the average expression of the receptor in one cluster and the average expression of the ligand in the other cluster, and the *P* value indicates the cell type specificity of the crosstalk.

### Microarray-based ST and statistical analysis

Microarray-based ST analysis was performed as previously described^28^. Xue et al. annotated 4 types of ST slides (epithelium, stroma, nerve, and others). We adopted this annotation for our analysis. Downstream analysis was performed with Seurat (v3.2.3) with default parameters, and joint clustering was performed with BayesSpace^54^. The Cell2location package^55^ was used for the deconvolution of T cells (75% was set as the cutoff), which was lower than the 25% cutoff reported by the other groups. The UCell package^56^ was used to score spots independently for enrichment in various MSigDB signatures. To determine the localization of basal-like PDAC cells and CD4-CCR7, CD4-FOXP3/CTLA-4, and CD4-FOXP3/BATF cells, we ultimately mapped the basal-like score to all epithelial areas.

### Cell culture

Cells were used within 10 passages for all experiments and were routinely confirmed to be mycoplasma-free during the course of the study (LT07-318, Lonza). KPC1412 cells were a gift from D. Tuveson (Cold Spring Harbor Laboratory). MIA PaCa-2 (ATCC, CRL-1420), BxPC-3 (ATCC, CRL-1687), PK1 (RCB1972), Hs 766 T (ATCC, HTB-134), SUIT-2 (JCRB1094), PANC-1 (ATCC, CRL-1469), and AsPC-1 (ATCC, CRL-1682) cells were cultured in Roswell Park Memorial Institute medium supplemented with 10% FBS and penicillin/streptomycin. HEK293 cells were cultured in Dulbecco’s modified Eagle’s medium supplemented with 10% FBS. The cell lines were purchased from commercial vendors, and their identities were validated by short tandem repeat analysis.

### Plasmids and stable cell lines

Dox-induced PKM1 and Dox-induced PKM2 plasmids were prepared as described in our previous paper^22^. Lentivirus particles were produced by co-transfection of lentiviral-expressing constructs with packaging plasmids (psPAX2 and pMD2.G) into HEK293 cells and then concentrated using a Lenti-X Concentrator (Takara, 631231). For lentiviral infection, 30% confluent pancreatic cancer cells were incubated with virus plus polybrene (TR1003G; Thermo Fisher Scientific) overnight. Puromycin (1 μg/mL) was added 48 h after infection. After 7 days, the cells were cultured in medium without puromycin.

### Orthotopic pancreatic cancer xenograft mouse model

For in vivo orthotopic transplantation, 10^6^ MIA PaCa-2 or 5 × 10^5^ KPC1412 cancer cells were injected into the pancreas of NU/J mice (strain 002019, the Jackson Laboratory) or C57/BL-6 mice (strain 000664, the Jackson Laboratory), respectively. Tumors were monitored by bioluminescence imaging (BLI). To perform BLI, D-luciferin was reconstituted as per the manufacturer’s protocol (Goldbio, LUCK-100) and administered intraperitoneally (100 μL; 15 mg/mL) 12 min before imaging with an IVIS Spectrum scanner. The mice were anesthetized with 1.5% to 2% isoflurane in the air during scanning. The amount of emitted light was quantified as “radiance (photons)”, p/sec/cm^2^/sr (steradian), using Living Image Software. The animals were first imaged on Day 3 after surgery and were excluded from the study if no tumors were present.

ASOs were systemically delivered by intraperitoneal injection at a dosage of 250 mg/kg/week with five consecutive daily injections, followed by 2 days of rest, for 2 or 3 weeks. The anti-mouse CTLA-4 antibody (Bioxcell, BP0164) and a mouse IgG2b isotype control (Bioxcell, BP0086) were delivered by intraperitoneal injection at a dosage of 100 μg every 3 days. All animal protocols were approved by the Institutional Animal Care and Use Committee of Cold Spring Harbor Laboratory.

### ASOs

TMOs were synthesized and purified as described previously^57^. PS-MOE and LNA ASOs were purchased from Integrated DNA Technologies (IDT). ASOs synthesized on a large scale for animal work were purified by high-performance liquid chromatography. We dissolved the ASOs in water and diluted them in saline before use or dissolved them in DPBS directly before use. A list of the oligonucleotide sequences and modifications is provided in Supplementary Table S2. The ASOs tested in C57/BL-6 mice were MOE16 (uniform MOE modification: 5′-ACTTGGTGAGCACGAT-3′) and mSCR2 (uniform MOE modification: 5′-TAACGTCCGTGAGTAG-3′).

### Transfection and free uptake of ASOs

Different amounts of ASOs, ranging from 1 to 100 nM, were delivered to MIA PaCa-2 and BxPC-3 cells using Lipofectamine 3000 transfection reagent (Invitrogen, L3000001) for 3 days, following the manufacturer’s instructions. Different amounts of mouse *Pkm* ASOs, ranging from 25 to 400 nM, were delivered to KPC1412 cells using Lipofectamine 3000 for 3 days. For free uptake, ASOs were directly added to the culture medium without a delivery reagent. After 3 and 5 days, the cell medium was replaced with fresh medium plus ASO, and the cells were harvested on Day 7. For siRNA transfection, cells were transfected with 50 nM siRNA, followed by RNA extraction on Day 2 and protein extraction on Day 3. Detailed information about the siRNAs is provided in Supplementary Table S3.

### Cell viability and proliferation assays

Pancreatic cancer cells were transfected with various concentrations of ASOs on Day 1. Then, 500 cells per well were plated in a 96-well plate on Day 2. Cell viability and growth were continuously measured using a cell-counting kit (CCK-8; MedChemExpress, HY-K0301) at the indicated time points. To calculate the IC50, cell viability was measured on Day 4, without continuous monitoring. For the free uptake experiments, 500 cells per well were plated in a 96-well plate on Day 1, after which 20 μM ASO was added. After 3 days, the cell medium was replaced with fresh medium plus a second dose of ASO. Cell viability was continuously measured at predetermined time points.

### PK activity assay and lactic acid production

PK activity was measured with a pyruvate kinase activity kit (ab83432; Abcam), and lactic acid production was measured with a L-Lactate Assay Kit (ab65330 and ab65331; Abcam) in accordance with the manufacturer’s instructions. Cells were either transfected with 50 nM ASO for 3 days or treated with 20 μM ASO for 5 days for free uptake and were then lysed in assay buffer. The lysates were deproteinized using a Deproteinizing Sample Preparation Kit (Abcam, ab204708).

### Flow cytometry

Cells (5 × 10^4^ cells/well) were seeded in six-well plates and treated with 20 μM ASO by free uptake for 7 days. The medium was replaced, and the ASO was replenished on Days 3 and 5. The cells were subsequently washed with PBS, fixed with 70% ice-cold ethanol, and incubated overnight at 4 °C. The cells were then washed with PBS, resuspended in Guava cell cycle reagent, and detected with a Guava flow cytometer (Luminex). The data were then analyzed using FlowJo software and fitted with ModFit software.

### RT-PCR

Cells or tissues were harvested at the experimental end points and homogenized with MagNA Lyser Green Beads (Roche, 03358941001). For RNA extraction, 1 mL of TRIzol (Invitrogen, 15596-018) was added to the homogenized tumor tissue or cells following the manufacturer’s protocol. RT-PCR was subsequently performed using ImProm-II Reverse Transcriptase (Promega, A3803) according to the manufacturer’s protocol. Radioactive PCR was conducted using ^32^P-α-dCTP and 1.25 U of AmpliTaq (N8080171; Invitrogen), with 35 amplification cycles. The radiolabeled PCR product was digested with PstI (R0140S, NEB) for 2 h at 37 °C to distinguish PKM1 (undigested) from PKM2 (cleaved into 2 bands). Products were separated on a 5% native polyacrylamide gel and then imaged with a Typhoon FAL7000 phosphorimager (GE HealthCare). For quantitative RT-PCR, a QuantStudio 6-flex Real-time PCR (Applied Biosystems) was used to analyze gene expression using gene-specific primers (Supplementary Table S1). The expression levels were normalized to those of *ACTB*. To calculate the PKM2/PKM1 ratio, we used the following equation: 2^–[(CELL^PKM2-PKM1^) – (HPNE^PKM2-PKM1^)].

### Western blotting assay

For protein extraction, cells or tissues were harvested and lysed on ice using RIPA lysis buffer containing protease inhibitor cocktail (Roche, 04693116001), followed by centrifugation at 12,000× *g* for 15 min at 4 °C. Then, the supernatant was collected, and the protein concentration was measured by a Bradford assay (Bio-Rad). Protein lysates were separated on an 8% to 16% precast SDS polyacrylamide gel (Bio-Rad), transferred onto a nitrocellulose membrane (Millipore), and incubated with anti-PKM2 (1:1000; Cell Signaling Technology, rabbit monoclonal, 4053S), anti-PKM1 (1:1000; Cell Signaling Technology, rabbit monoclonal, 7067S), anti-PKM1/2 (1:1000; Cell Signaling Technology, rabbit monoclonal, 3190S), anti-β-actin (1:1000; Sigma‒Aldrich, mouse monoclonal, A1978) or anti-vinculin (1:1000; Santa Cruz Bio, mouse monoclonal, sc-73614) antibodies overnight at 4 °C. Next, the membranes were incubated with 800CW goat anti-rabbit IgG secondary antibody (1:10000; LI-COR Biosciences, 926-32211) or 680RD goat anti-mouse IgG secondary antibody (1:10000; LI-COR Biosciences, 926-68070), detection was performed with an Odyssey Classic Infrared Imaging System, and the data were quantified with ImageJ software.

### Analysis of public datasets

The FASTQ files in the GSE211155 dataset were retrieved using Kingfisher. Raw Illumina sequencing reads were aligned to the genome assembly GRCh38 using STAR v2.5.3a. RSEM v1.3.0^58^ was used to extract counts per gene. The ∆PSI and FDR values of splicing changes were produced using rMATS turbo v4.3.0 (https://github.com/Xinglab/rmats-turbo) with default parameters and visualized with rmats2sashimiplot (https://github.com/Xinglab/rmats2sashimiplot)^59^. To explore potential differences in splicing regulators, we downloaded subtype information from the Yee laboratory (https://github.com/jjyeh) and RNA-seq data from UCSC. The pheatmap package (v 1.0.13, https://cran.r-project.org/web/packages/pheatmap) was used for visualization.

### Statistical analyses

Statistical tests were performed using GraphPad Prism software. All graphs plot individual data points to indicate the *n* values for each treatment and genotype, with the horizontal lines representing the mean ± SD. *P* values are reported as *P < 0.05; **P < 0.01; ***P < 0.001.

## Supplementary information

Supplementary Fig.S1

Supplementary Fig.S2

Supplementary Fig.S3

Supplementary Fig.S4

Supplementary Fig.S5

Supplementary Fig.S6

Supplementary Fig.S7

Supplementary Fig.S8

Supplementary Fig.S9

Supplementary Table S1

Supplementary Table S2

Supplementary Table S3

## Data Availability

All data associated with this study are presented in the paper or Supplementary Information. PDAC clinical samples are from Ruijin Hospital, Shanghai Jiao Tong University School of Medicine. scRNA-seq data used in this study are deposited in Gene Expression Omnibus (GEO) under accession code GSE202742 and the National Omics Data Encyclopedia (NODE) under accession code OEP003254. ST data used in this study are deposited in GEO under accession code GSE202740.
