# Supplementary material for: ASO-based PKM splice-switching therapy increases anti-CTLA-4 antibody efficacy in pancreatic ductal adenocarcinoma
Source: Cell Discov. 2026 Apr 21;12:28. doi: 10.1038/s41421-026-00882-9 (PMC13096517; doi:10.1038/s41421-026-00882-9)
Supplement: Supplementary file 2 — Supplementary Fig.S2 [file 41421_2026_882_MOESM2_ESM.pdf]

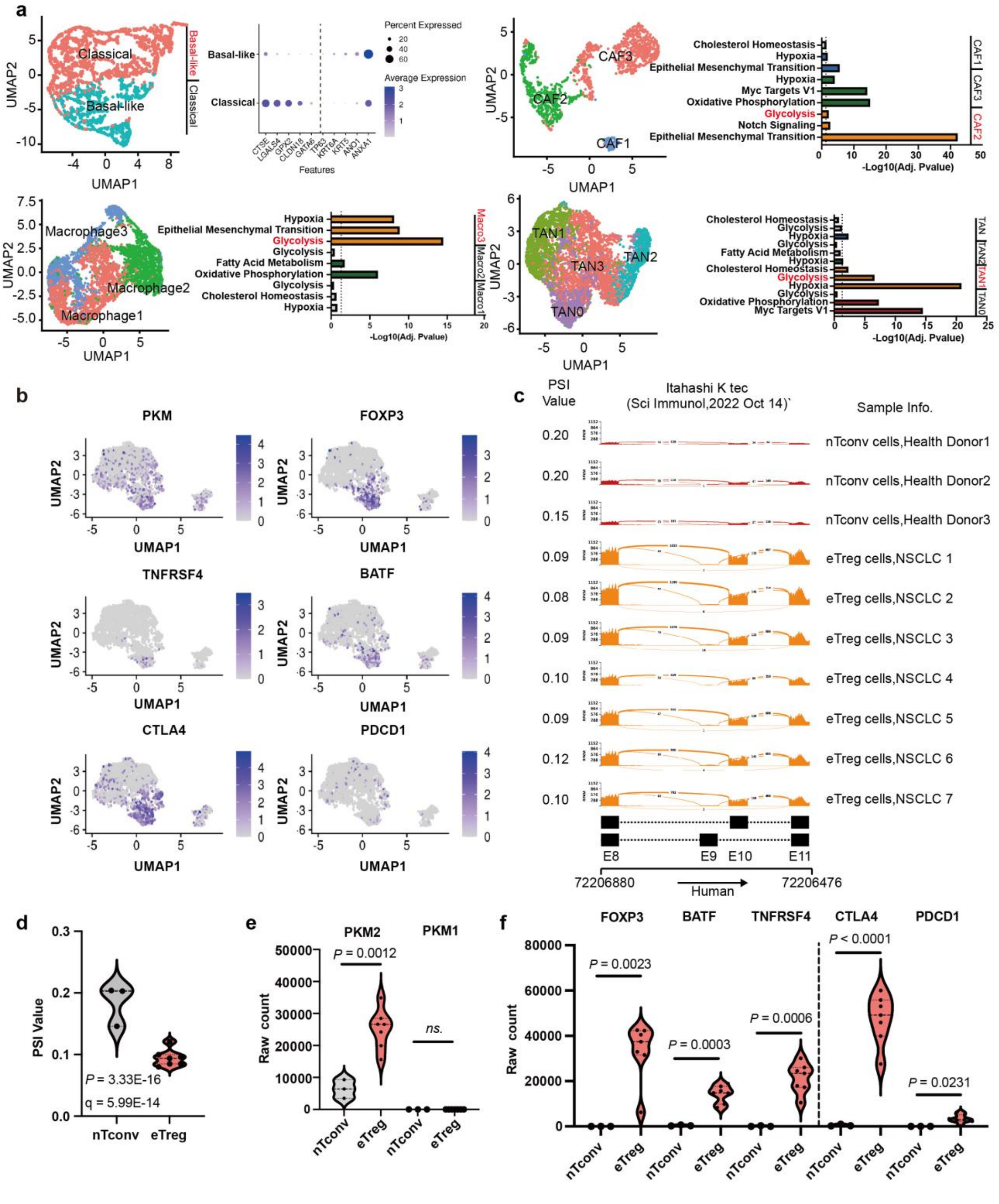

Supplementary Fig. S2 Broad screen for potential cells that can be targeted by PKM-ASO in the PDAC TME. a, Subcluster of scRNAseq, and GO analysis. **b**, *PKM*, *FOXP3*, *TNFRSF4*, *BATF*, *CTLA44*, and *PDCD1* expression along T_{reg} differentiation. **c**, Re-analysis of bulk RNAseq data of nT_{conv} (CD4-CCR7) and eT_{reg} (CD4-FOXP3/BATF) from public dataset GSE211155. rMATS was used for PSI calculation and statistical analysis, and

JCEC value was input into rMATSsashimiplot for visualization. **d**, PSI value of exon 9 (nT_{conv} = 3, eT_{reg} = 8). **e**, PKM2 was more highly expressed than PKM1 in eT_{reg}. Statistical analysis: unpaired two-sided t-test. **f,** Three markers of CD4-FOXP3/BATF cells from scRNAseq were highly expressed in eT_{reg}, and two immune checkpoint markers are also shown. Statistical analysis: unpaired two-sided t-test (c, d, e); Benjamini-Hochberg method to correct for multiple hypotheses testing (a); Log-rank Mantel–Cox test (g).
