# Supplementary material for: ASO-based PKM splice-switching therapy increases anti-CTLA-4 antibody efficacy in pancreatic ductal adenocarcinoma
Source: Cell Discov. 2026 Apr 21;12:28. doi: 10.1038/s41421-026-00882-9 (PMC13096517; doi:10.1038/s41421-026-00882-9)
Supplement: Supplementary file 3 — Supplementary Fig.S3 [file 41421_2026_882_MOESM3_ESM.pdf]

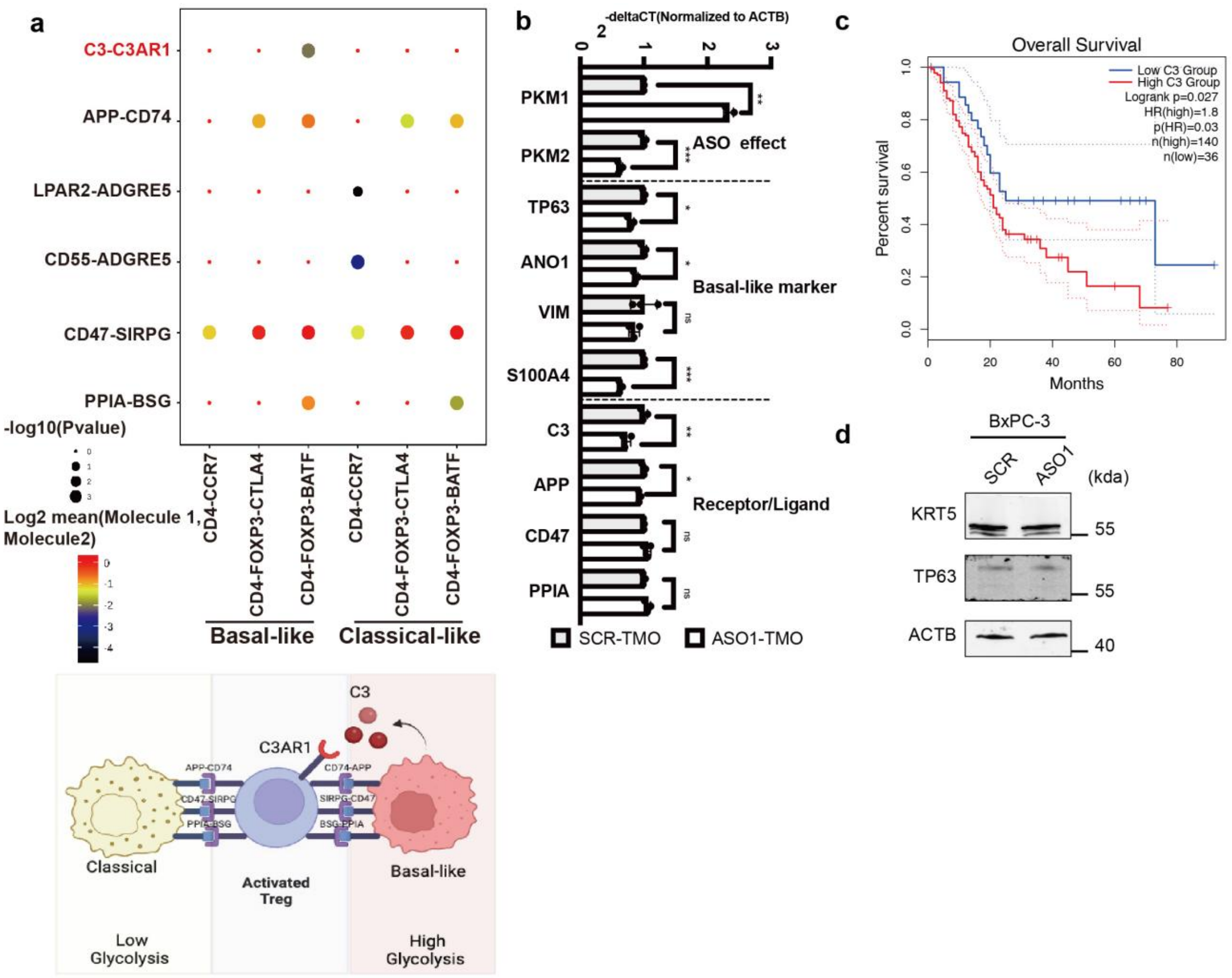

Supplementary Fig. S3 ASO-mediated *PKM* splicing modulation suppresses C3 expression and basal-like PDAC signaling to T_{regs}. a. CellPhoneDB analysis shows potential receptor-ligand pairs for CD4-FOXP3/BATF chemotaxis. Schematic of CellPhoneDB. C3 was secreted by basal-like PDAC cells. C3-C3AR1 interaction is seen only between basal-like cells and activated T_{reg}. **b**, The basal-like cell line BxPC-3 was transfected with 100 nM SCR-TMO or ASO1-TMO. RNA was extracted after 48 h and analyzed by RT-qPCR. **c**, Kaplan–Meier survival analysis for low and high C3 in PDAC patients from TCGA. **d**, Western blot of KRT5 and TP63 proteins after 72 h transfection with 100 nM SCR-TMO or ASO1-TMO.
