# Supplementary material for: ASO-based PKM splice-switching therapy increases anti-CTLA-4 antibody efficacy in pancreatic ductal adenocarcinoma
Source: Cell Discov. 2026 Apr 21;12:28. doi: 10.1038/s41421-026-00882-9 (PMC13096517; doi:10.1038/s41421-026-00882-9)
Supplement: Supplementary file 4 — Supplementary Fig.S4 [file 41421_2026_882_MOESM4_ESM.pdf]

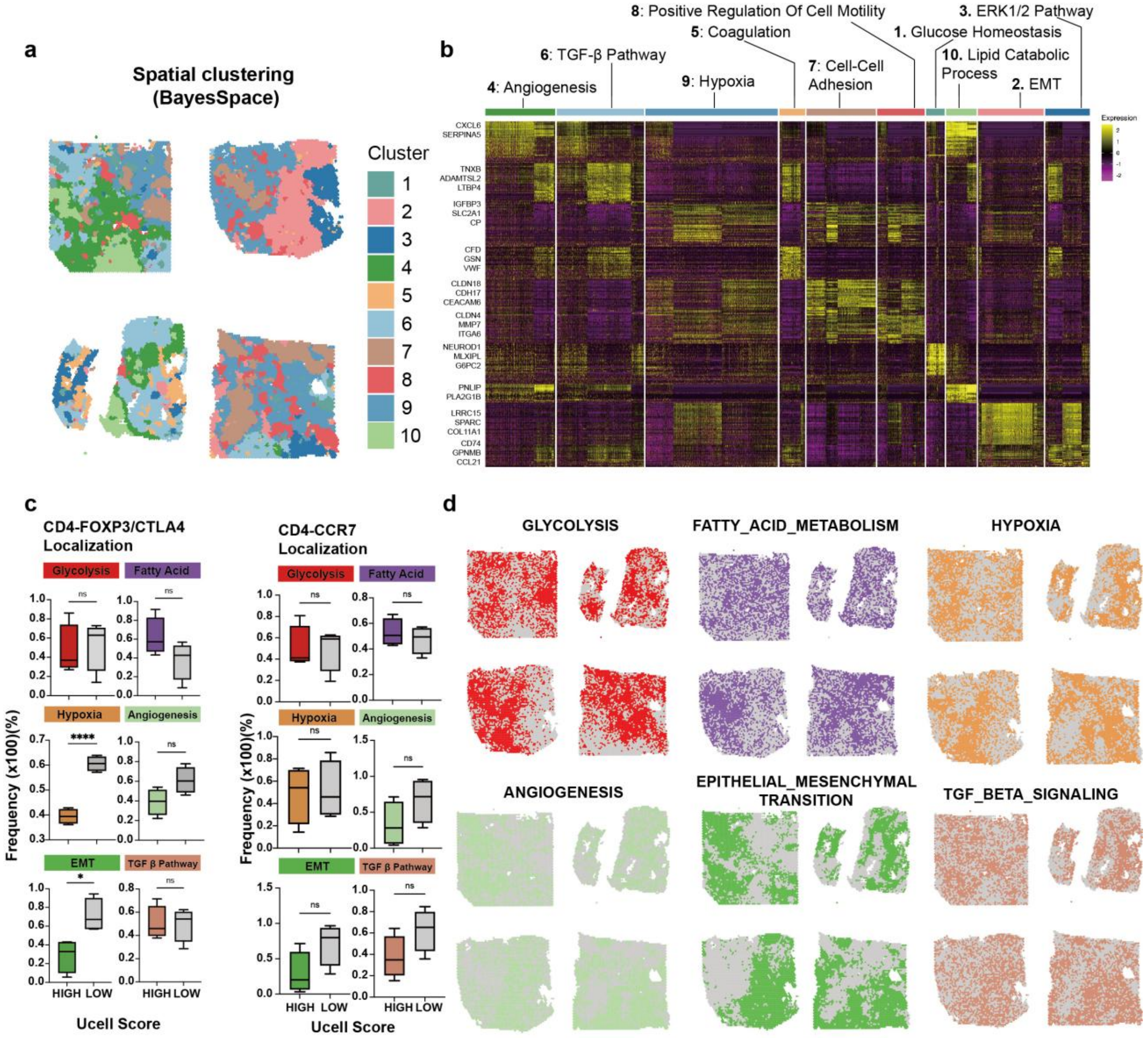

Supplementary Fig. S4. Microarray-based spatial transcriptomics reveals that basal-like tumor cells are surrounded by CD4-FOXP3/BATF cells. a, Ten spatial clusters retrieved from BayesSpace. **b**, GO analysis of each cluster by EnrichR. **c**, Frequency of CD4-FOXP3/CTLA-4 (left) and CD4-CCR7 (right) cells localized in high/low score areas (n = 4 biological replicates). CD4-FOXP3/CTLA-4 and CD4-CCR7 cells do not show significant glycolysis metabolism and show low EMT, compared to CD4-FOXP3/BATF cells. **d,** UCell was used for evaluating the signature score of each spot in each section; 50 % was used as cutoff to define high and low areas. Statistical analysis: unpaired two-sided t-test (c).
