# Supplementary material for: ASO-based PKM splice-switching therapy increases anti-CTLA-4 antibody efficacy in pancreatic ductal adenocarcinoma
Source: Cell Discov. 2026 Apr 21;12:28. doi: 10.1038/s41421-026-00882-9 (PMC13096517; doi:10.1038/s41421-026-00882-9)
Supplement: Supplementary file 5 — Supplementary Fig.S5 [file 41421_2026_882_MOESM5_ESM.pdf]

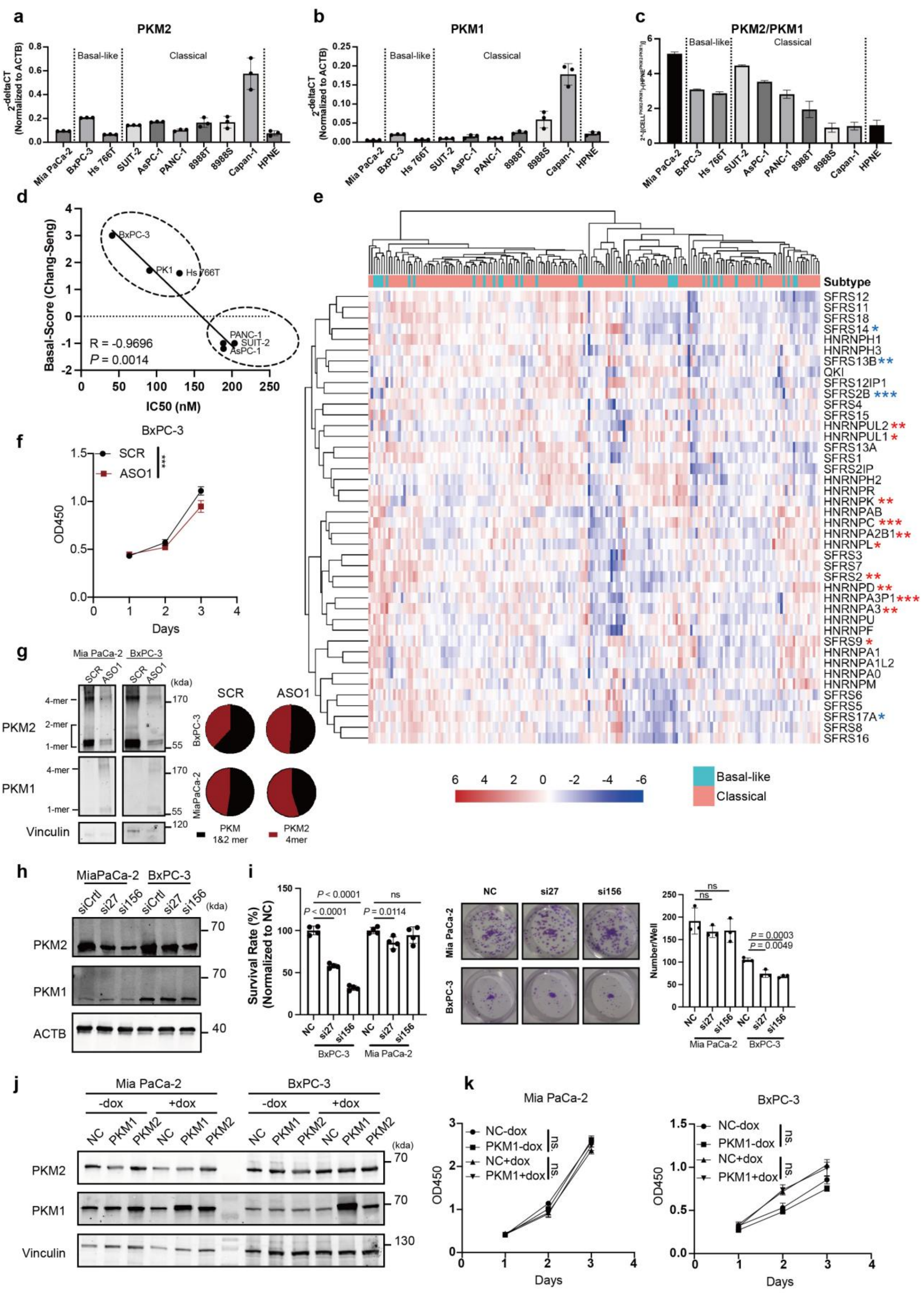
a
PKM2
2^-deltaCT (Normalized to ACTB)
Basal-like
Classical
Mia PaCa-2
BxPC-3
Hs 766T
SUIT-2
AsPC-1
PANC-1
8988T
8988S
Capan-1
HPNE
b
PKM1
c
PKM2/PKM1
d
Basal-Score (Chang-Seng)
BxPC-3
PK1
Hs 766T
PANC-1
SUIT-2
AsPC-1
R = -0.9696
P = 0.0014
IC50 (nM)
e
Subtype
SFRS12
SFRS11
SFRS18
SFRS14 *
HNRNPH1
HNRNPH3
SFRS13B **
QKI
SFRS12IP1
SFRS2B ***
SFRS4
SFRS15
HNRNPUL2 **
HNRNPUL1 *
SFRS13A
SFRS1
SFRS2IP
HNRNPH2
HNRNPR
HNRNPK **
HNRNPAB
HNRNPC ***
HNRNPA2B1 **
HNRNPL *
SFRS3
SFRS7
SFRS2 **
HNRNPD **
HNRNPA3P1 ***
HNRNPA3 **
HNRNPU
HNRNPF
SFRS9 *
HNRNPA1
HNRNPA1L2
HNRNPA0
HNRNPM
SFRS6
SFRS5
SFRS17A *
SFRS8
SFRS16
Basal-like
Classical
f
BxPC-3
SCR
ASO1
***
OD450
Days
g
Mia PaCa-2
BxPC-3
SCR
ASO1
(kda)
4-mer
2-mer
1-mer
PKM2
PKM1
Vinculin
170
55
120
SCR
ASO1
BxPC-3
MiaPaCa-2
PKM 1&2 mer
PKM2 4mer
h
MiaPaCa-2
BxPC-3
siCrtl
si27
si156
(kda)
PKM2
PKM1
ACTB
70
70
40
i
Survival Rate (%) (Normalized to NC)
P < 0.0001
P < 0.0001
ns
P = 0.0114
NC
si27
si156
BxPC-3
Mia PaCa-2
Mia PaCa-2
BxPC-3
Number/Well
ns
ns
P = 0.0003
P = 0.0049
j
Mia PaCa-2
BxPC-3
-dox
+dox
NC
PKM1
PKM2
(kda)
PKM2
PKM1
Vinculin
70
70
130
k
Mia PaCa-2
BxPC-3
NC-dox
PKM1-dox
NC+dox
PKM1+dox
ns.
OD450
Days

Supplementary Fig. S5. Transfection of ASO1-TMO induces *PKM* splice switching in PDAC cells. a, PKM2 expression in PDAC cell lines. HPNE was originally derived from normal pancreas ductal epithelium. **b**, PKM1 expression in PDAC cell lines. **c**, PKM2/PKM1 expression in PDAC cell lines, normalized to HPNE. **d**, Pearson correlation of IC50 and basal-like score. **e**, Splicing-factor expression differences between basal-like and classical PDAC patients, based on PAAD-TCGA (Red, upregulated; Blue, downregulated). **f**, Cell viability assays for BxPC-3 cell line at each time point. Cells were transfected with 50 nM ASO on day 0, 0.5×10^3 cells were plated on a 96-well plate on day 1, and the OD450 was monitored daily by a colorimetric assay. Statistical analysis: two-way ANOVA. **g**, Western blot of PKM2 and PKM1, 72 h after 50 nM ASO transfection. Cells were crosslinked with disuccinimidyl suberate (DSS) for 30 min and then lysed in RIPA buffer. The proportion of PKM2 monomer, dimer, and tetramer was quantified by ImageJ. **h**, Western blot of PKM2 and PKM1, 72 h after transfection with PKM2-specific siRNA (50 nM). **i**, Survival rate of BxPC-3 and MIA PaCa-2 cells on day 3 after transfection with siRNA (50 nM), and colony formation on day 7. **j**, Western blot of PKM2 and PKM1 after treatment with 1 µg/mL Doxycycline for 1 day. **k**, Cell viability assays at each time point, for MIA PaCa-2 (left) and BxPC-3 (right). Fresh medium with 1 µg/mL Doxycycline was replaced every day. Statistical analysis: unpaired two-sided t-test (e, i); Two-way ANOVA (f, k); Pearson correlation analysis (d). *$P < 0.05$, **$P<0.01$, ***$P<0.001$.
