# Supplementary material for: ASO-based PKM splice-switching therapy increases anti-CTLA-4 antibody efficacy in pancreatic ductal adenocarcinoma
Source: Cell Discov. 2026 Apr 21;12:28. doi: 10.1038/s41421-026-00882-9 (PMC13096517; doi:10.1038/s41421-026-00882-9)
Supplement: Supplementary file 6 — Supplementary Fig.S6 [file 41421_2026_882_MOESM6_ESM.pdf]

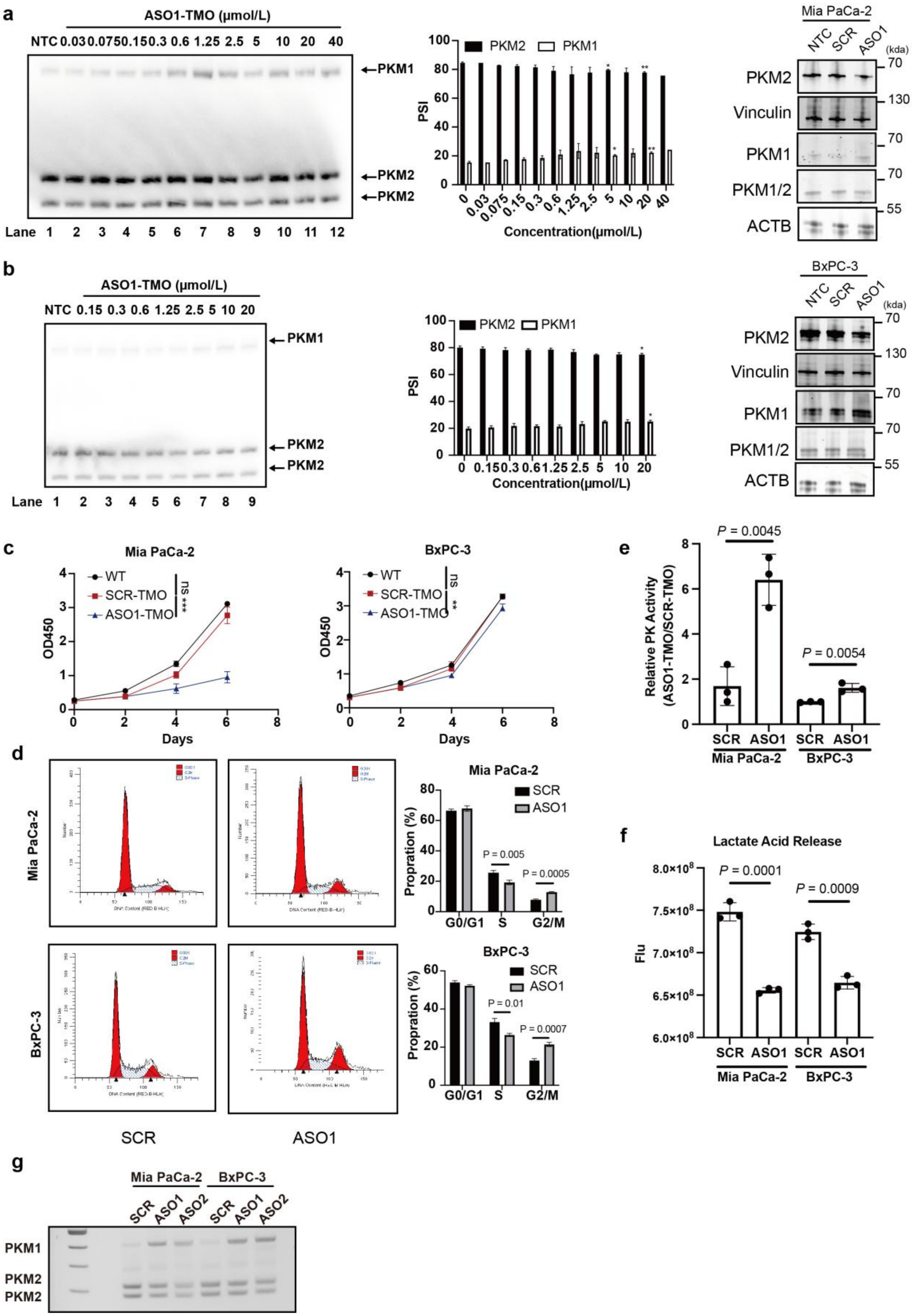

a
ASO1-TMO (μmol/L)
NTC 0.03 0.075 0.15 0.3 0.6 1.25 2.5 5 10 20 40
PKM1
PKM2
PKM2
Lane 1 2 3 4 5 6 7 8 9 10 11 12
PKM2 PKM1
PSI
Concentration(μmol/L)
Mia PaCa-2
NTC SCR ASO1
(kda)
PKM2
Vinculin
PKM1
PKM1/2
ACTB
b
ASO1-TMO (μmol/L)
NTC 0.15 0.3 0.6 1.25 2.5 5 10 20
Lane 1 2 3 4 5 6 7 8 9
BxPC-3
c
Mia PaCa-2
BxPC-3
WT
SCR-TMO
ASO1-TMO
ns
OD450
Days
d
SCR
ASO1
Propration (%)
G0/G1 S G2/M
P = 0.005
P = 0.0005
P = 0.01
P = 0.0007
e
P = 0.0045
P = 0.0054
Relative PK Activity (ASO1-TMO/SCR-TMO)
f
Lactate Acid Release
P = 0.0001
P = 0.0009
Flu
g
Mia PaCa-2 BxPC-3
SCR ASO1 ASO2 SCR ASO1 ASO2

Supplementary Fig. S6 Delivery of ASO-TMO by free uptake induces *PKM* splice switching in PDAC cells. a, ASO1-TMO induces *PKM* splice switching in a dose-dependent manner. Radioactive RT-PCR analysis (left) after treating MIA PaCa-2 cells with varying ASO1-TMO concentrations by free uptake for 7 days. Fresh medium and ASO were changed on days 3 and 5. ImageJ was used for quantification (middle). Western blot of PKM2, PKM1, and total PKM expression. **b**, Same as (a) but for BxPC-3 cells. **c**, ASO1-TMO slows down growth of MIA PaCa-2 and BxPC-3 cells. The cells were treated with 20 µmol/L ASO by free uptake for the indicated times, and the OD450 was monitored daily by colorimetric assay. **d**, Representative cell-cycle analysis by propidium iodide DNA staining of MIA PaCa-2 and BxPC-3 cells treated with 20 µmol/L ASO by free uptake for 7 days. **e**, PK activity of MIA PaCa-2 and BxPC-3 cells after treating with ASO as in (a) and (b). **f**, Lactic acid was measured after treating MIA PaCa-2 and BxPC-3 cells with ASO as in (a) and (b). **g**, ASO2-TMO, with a different *PKM* target sequence, showed comparable efficacy to ASO1-TMO. Statistical analysis: unpaired two-sided t-test (a, b, e, d, f); two-way ANOVA (c).
