# Supplementary material for: ASO-based PKM splice-switching therapy increases anti-CTLA-4 antibody efficacy in pancreatic ductal adenocarcinoma
Source: Cell Discov. 2026 Apr 21;12:28. doi: 10.1038/s41421-026-00882-9 (PMC13096517; doi:10.1038/s41421-026-00882-9)
Supplement: Supplementary file 7 — Supplementary Fig.S7 [file 41421_2026_882_MOESM7_ESM.pdf]

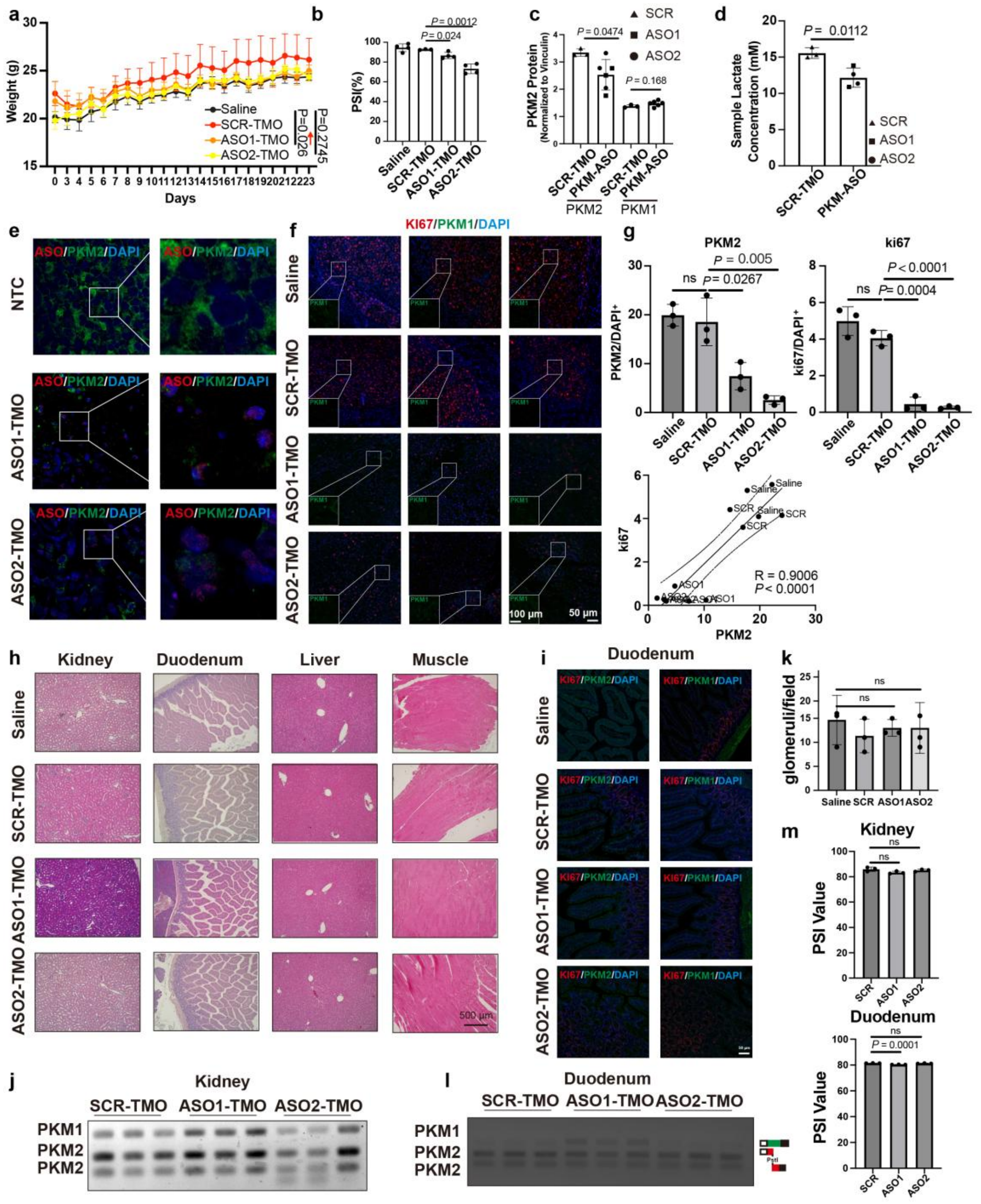

Supplementary Fig. S7 ASO1-TMO and ASO2-TMO inhibit MIA PaCa-2 cell growth in a xenograft model. a, Mice were weighed every day. **b**, Quantification of *PKM* splice switching in Figure 6f. **c**, Quantification of PKM1 and PKM2 in Figure 6h, normalized to Vinculin. **d**, Lactic acid concentration in samples prepared from tumors from mice treated with SCR-TMO, ASO1-TMO, or ASO2-TMO. **e**, Representative IF images of tumor sections for PKM2 (green) or ASO antibody (red) and nuclei with DAPI (blue) from the same tumor samples as in panel d. Scale bar, 50 µm. **f**, Representative IF images of tumor sections for PKM1 (green) or Ki67 proliferation marker (red) and nuclei (blue) from the same tumor samples as in panel d. Scale bar, 50 µm. **g**, Summary data from Fig. 5i**)** of Ki67^{+}

and PKM2$^+$ cell numbers normalized to DAPI$^+$ nuclei for each treatment group (n = 5 random fields). **h**, Representative H&E images of kidney, duodenum, liver, and muscle from the same animals as in panel d. Scale bar, 500 µm. **i**, Representative IF images of duodenum section for PKM2 (green) or proliferation marker (Ki67, red) and nuclei (blue) from the same animals as in panel d. Scale bar, 50 µm. **j-l**, RT-PCR and quantification of *PKM* splicing in kidney and duodenum. **k**, glomeruli per field from the same animals as in panel d. Statistical analysis: unpaired two-sided t-test (b, c, d, g, k, m); two-way ANOVA (a); Pearson correlation analysis (g).
