# Supplementary material for: ASO-based PKM splice-switching therapy increases anti-CTLA-4 antibody efficacy in pancreatic ductal adenocarcinoma
Source: Cell Discov. 2026 Apr 21;12:28. doi: 10.1038/s41421-026-00882-9 (PMC13096517; doi:10.1038/s41421-026-00882-9)
Supplement: Supplementary file 8 — Supplementary Fig.S8 [file 41421_2026_882_MOESM8_ESM.pdf]

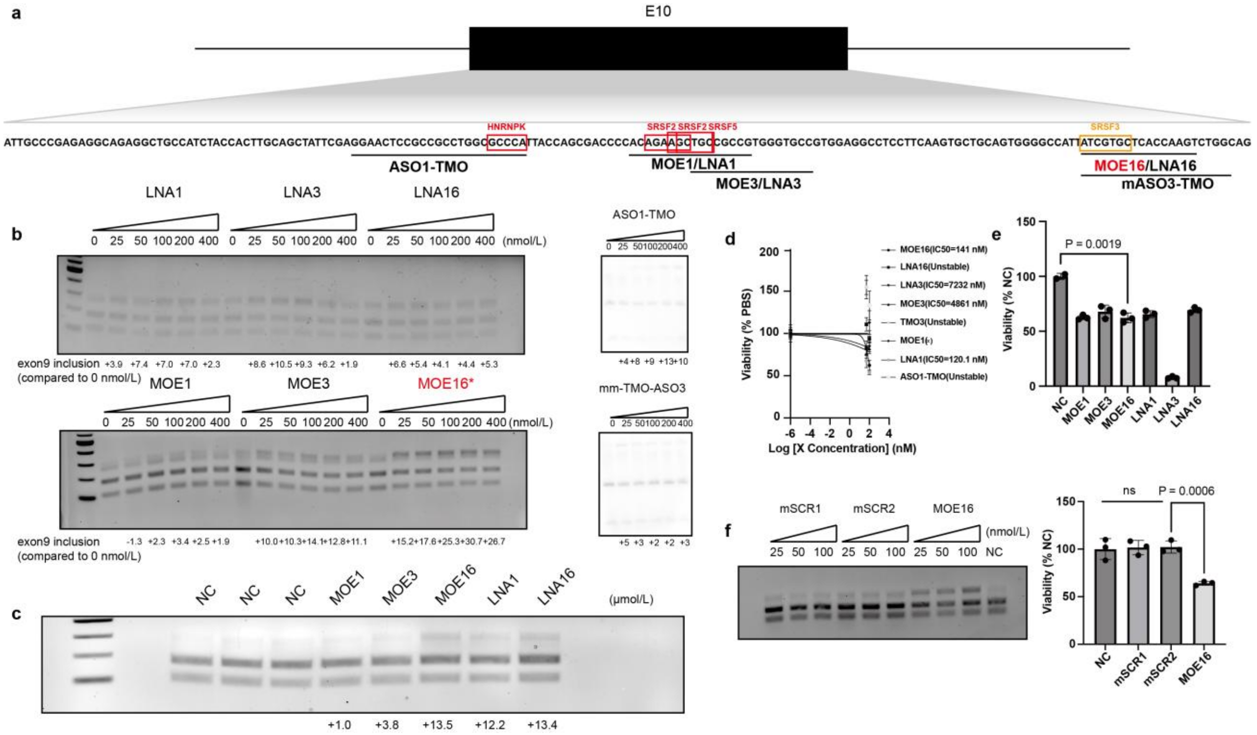

Supplementary Fig. S8 Screening approach to identify MOE16 for administration to C57BL6 mice. a, Schematic of five ASOs targeting *Pkm* exon 10 (upper case). Each ASO has uniform MOE, mixed LNA/DNA, or TMO modifications (Supplementary Table S2). ASOs affect *Pkm* alternative splicing by occupying the binding sites on the pre-mRNA of one or more splicing factors. Predicted RNA-binding motifs along *Pkm* exon 10 using RBPmap and Splicead as described[1-3]. **b**, Radioactive RT-PCR shows the extent of *Pkm* splice switching after transfecting KPC1412 cells with the indicated concentrations of ASO for 2 days. ImageJ was used for quantification of Pkm1 and Pkm2 isoforms (bottom). **c**, Radioactive RT-PCR shows the extent of *Pkm* splice switching after ASO treatment via free uptake for 6 days. LNA3 was not included, due to cell toxicity. **d**, IC50 of each ASO. **e**, Viability of KPC1412 cells 3 days after ASO transfection. **f**, Splice switching and toxicity of the mouse ASO control. Statistical analysis: unpaired two-sided t-test (e, f).

References

1 Paz, I., Kosti, I., Ares, M., Jr., Cline, M. & Mandel-Gutfreund, Y. RBPmap: a web server for mapping binding sites of RNA-binding proteins. Nucleic Acids Res 42, W361-367 (2014). https://doi.org:10.1093/nar/gku406

2 Piva, F., Giulietti, M., Nocchi, L. & Principato, G. SpliceAid: a database of experimental RNA target motifs bound by splicing proteins in humans. Bioinformatics 25, 1211-1213 (2009). https://doi.org:10.1093/bioinformatics/btp124

3 Wang, Z., Jeon, H. Y., Rigo, F., Bennett, C. F. & Krainer, A. R. Manipulation of PK-M mutually exclusive alternative splicing by antisense oligonucleotides. Open Biol 2, 120133 (2012). https://doi.org:10.1098/rsob.120133
