# Supplementary material for: ASO-based PKM splice-switching therapy increases anti-CTLA-4 antibody efficacy in pancreatic ductal adenocarcinoma
Source: Cell Discov. 2026 Apr 21;12:28. doi: 10.1038/s41421-026-00882-9 (PMC13096517; doi:10.1038/s41421-026-00882-9)
Supplement: Supplementary file 9 — Supplementary Fig.S9 [file 41421_2026_882_MOESM9_ESM.pdf]

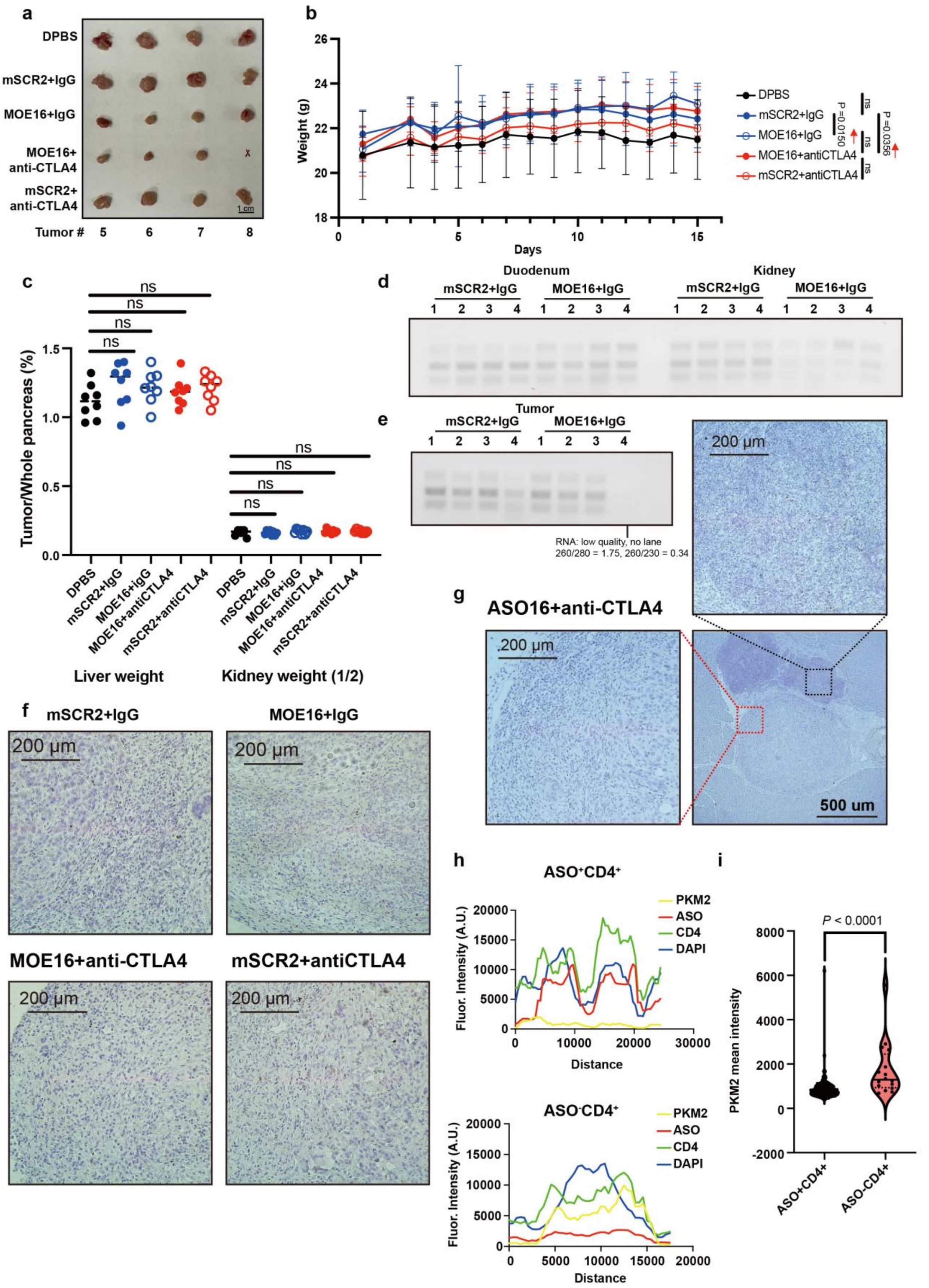
a
DPBS
mSCR2+IgG
MOE16+IgG
MOE16+
anti-CTLA4
mSCR2+
anti-CTLA4
1 cm
Tumor #
5
6
7
8
b
Weight (g)
Days
DPBS
mSCR2+IgG
MOE16+IgG
MOE16+antiCTLA4
mSCR2+antiCTLA4
ns
P=0.0150
ns
P=0.0356
ns
c
Tumor/Whole pancreas (%)
ns
DPBS
mSCR2+IgG
MOE16+IgG
MOE16+antiCTLA4
mSCR2+antiCTLA4
Liver weight
Kidney weight (1/2)
d
Duodenum
Kidney
mSCR2+IgG
MOE16+IgG
e
Tumor
RNA: low quality, no lane
260/280 = 1.75, 260/230 = 0.34
200 µm
f
mSCR2+IgG
MOE16+IgG
MOE16+anti-CTLA4
mSCR2+antiCTLA4
200 µm
g
ASO16+anti-CTLA4
200 µm
500 um
h
ASO⁺CD4⁺
PKM2
ASO
CD4
DAPI
Fluor. Intensity (A.U.)
Distance
ASO⁻CD4⁺
i
P < 0.0001
PKM2 mean intensity
ASO+CD4+
ASO-CD4+

Supplementary Fig. S9 ASO-based *Pkm* splice switching enhances anti-CTLA-4 response in PDAC. a, Tumor weight/weight of whole pancreas on day 15 (n = 4 per group). **b**, Mice weights during treatment. **c**, Weight of liver and kidney (halves) when harvesting tumors (n = 8). **d**, *Pkm* splice switching in duodenum and kidney 15 days after i.p. injection of ASO. **e**, *Pkm* splice switching in tumors 15 days after i.p. injection of ASO. **f**, T_{reg} cells surrounding the tumor area. Scale bars, 200 μm (inset). **g**, T_{reg} cell infiltration in tumor #4, corresponding to Fig. 8 i. **h**, Profile intensities of PKM2, ASO, and DAPI in $CD4^{+}ASO^{+}$ cells (white line) and $CD4^{+}ASO^{-}$ cells (red line). **i**, PKM2 intensity in $CD4^{+}ASO^{+}$ cells and $CD4^{+}ASO^{-}$ cells. Statistical analysis: unpaired two-sided t-test (c, i); two-way ANOVA (b).
