# Supplementary material for: ASO-based PKM splice-switching therapy increases anti-CTLA-4 antibody efficacy in pancreatic ductal adenocarcinoma
Source: Cell Discov. 2026 Apr 21;12:28. doi: 10.1038/s41421-026-00882-9 (PMC13096517; doi:10.1038/s41421-026-00882-9)
Supplement: Supplementary file 10 — Supplementary Table S1 [file 41421_2026_882_MOESM10_ESM.pdf]

Supplementary Table S1 Primers

PRIMER NAME	Sequence（5‘-3’）
TP63-R	AGGACACGTCGAAACTGTGC
TP63-F	GGACCAGCAGATTCAGAACGG
S100A4-R	CTGGGCTGCTTATCTGGGAAG
S100A4-F	GATGAGCAACTTGGACAGCAA
ANO1-R	AGGGCCTCTTGTGATGGTACA
ANO1-F	CTGATGCCGAGTGCAAGTATG
VIM-R	CTTTGTCGTTGGTTAGCTGGT
VIM-F	GACGCCATCAACACCGAGTT
PKM1-R	GCTGCCTCAGCCTCACGAGC
PKM1-F	CCCACTCGGGCTGAAGGCAGTG
PKM2-R	GGCAGCCTCTGCCTCACGGG
PKM2-F	CCCACTCGGGCTGAAGGCAGTG
ACTB-R	CTCCTTAATGTCACGCACGAT
ACTB-F	CATGTACGTTGCTATCCAGGC
C3-R	GGAAGTCGTGGACAGTAACAG
C3-F	GGGGAGTCCCATGTACTCTATC
APP-R	AGAAGGGCATCACTTACAAACTC
APP-F	CAAGCAGTGCAAGACCCATC
CD47-R	CTCATCCATACCACCGGATCT
CD47-F	AGAAGGTGAAACGATCATCGAGC
PPIA-R	GGACCCGTATGCTTTAGGATGA
PPIA-F	CCCACCGTGTTCTTCGACATT
OligodT	TTTTTTTTTTTTTTTN
Human-Hotgel-PKM-R：	CATTCATGGCAAAGTTCACC
Human-Hotgel-PKM-F	AGAAACAGCCAAAGGGGACT
Mouse-Hotgel-PKM-F	AAACAGCCAAGGGGGACTAC
Mouse-Hotgel-PKM-R	CGAGCAGTCTGGGGATTTCG
