# Supplementary material for: ASO-based PKM splice-switching therapy increases anti-CTLA-4 antibody efficacy in pancreatic ductal adenocarcinoma
Source: Cell Discov. 2026 Apr 21;12:28. doi: 10.1038/s41421-026-00882-9 (PMC13096517; doi:10.1038/s41421-026-00882-9)
Supplement: Supplementary file 11 — Supplementary Table S2 [file 41421_2026_882_MOESM11_ESM.pdf]

Supplementary Table S2 ASOs

**ASO name**	**Sequence 5' – 3'**	**Chemistry**
ASO1-TMO	AGGCGGCGGAGTTCCTCA	pppppppppppppppppd
ASO2-TMO	GTGAGGACGATTATGGCC	pppppppppppppppppd
SCR-TMO	GTTGCATACGCGAGGCGC	pppppppppppppppppd
mASO3-TMO	CCAGACTTGGTGAGCACGAT	pppppppppppppppppppd
mSCR1	ACGTCCGTGAGTAGGC	Uniform MOE/PS
mSCR2	TAACGTCCGTGAGTAG	Uniform MOE/PS
MOE1	CGGCGGCAGCTTCTGT	Uniform MOE/PS
MOE3	GGCACCCACGGCGGCA	Uniform MOE/PS
MOE16	ACTTGGTGAGCACGAT	Uniform MOE/PS
LNA1	CGGCGGCAGCTTCTGT	+dd+dd+dd+dd+dd+
LNA3	GGCACCCACGGCGGCA	+dd+dd+dd+dd+dd+
LNA16	ACTTGGTGAGCACGAT	+dd+dd+dd+dd+dd+

*p = thiophosphoramidate morpholino (TMO); d = DNA base; all ASOs have uniform PS backbone and 5-methyl-C modifications; + = Locked nucleic acid (LNA). For in vitro work, ASOs were dissolved in water and diluted in saline before use. For in vivo work, ASOs were either dissolved in water and diluted in saline before use, or dissolved directly in DPBS.
