# Supplementary material for: ASO-based PKM splice-switching therapy increases anti-CTLA-4 antibody efficacy in pancreatic ductal adenocarcinoma
Source: Cell Discov. 2026 Apr 21;12:28. doi: 10.1038/s41421-026-00882-9 (PMC13096517; doi:10.1038/s41421-026-00882-9)
Supplement: Supplementary file 12 — Supplementary Table S3 [file 41421_2026_882_MOESM12_ESM.pdf]

## Supplementary Table S3 siRNAs

| siRNA | Sense 5'-3' | Antisense 5'-3' |
|---|---|---|
| siCtrl | AGGCAGAGGCUGCCAUCUAUU | UAGAUGGCAGCCUCUGCCUUU |
| si27 | CCAUAAUCGUCCUCACCAAUU | UUGGUGAGGACGAUUAUGGUU |
| si156 | CUUACGCUGAGUACUUCGAUU | UCGAAGUACUCAGCGUAAGUU |
