# Supplementary material for: ASO-based PKM splice-switching therapy increases anti-CTLA-4 antibody efficacy in pancreatic ductal adenocarcinoma
Source: Cell Discov. 2026 Apr 21;12:28. doi: 10.1038/s41421-026-00882-9 (PMC13096517; doi:10.1038/s41421-026-00882-9)
Supplement: Supplementary file 1 — Supplementary Fig.S1 [file 41421_2026_882_MOESM1_ESM.pdf]

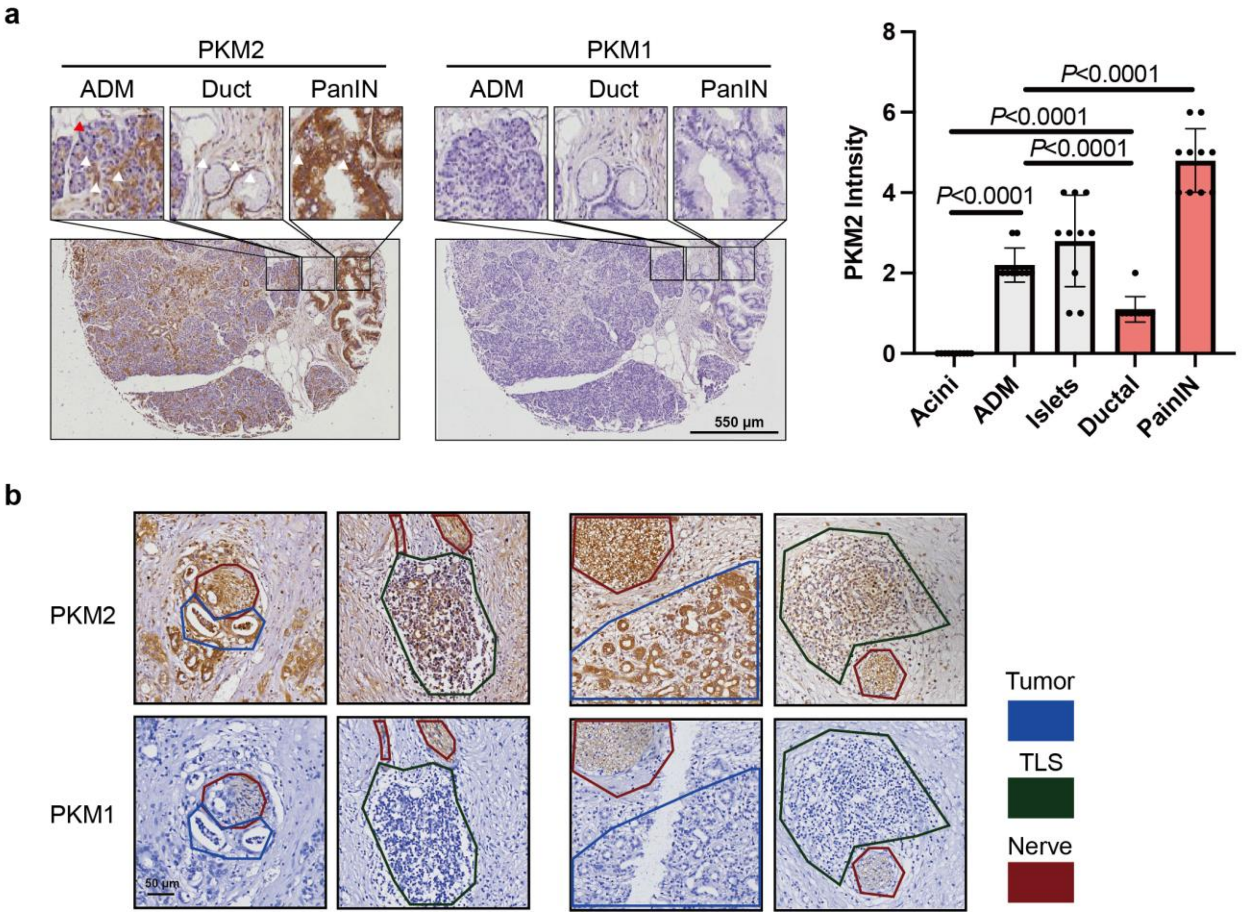

Supplementary Fig. S1. Expression and distribution of PKM1/2 in the PDAC TME, and clinical significance. **a**, IHC staining of PKM2 and PKM1(left) and intensity score (right) of PKM2 in PDAC patient samples. Insets show higher magnification of normal acinar cells, duct cells, ADM, and PanIN. Scale bars, 550 µm. For quantification of intensity, n = 10 per group. Statistical analysis: unpaired two-sided t-test. **b**, Structures were manually annotated with Qupath: tumor (blue), TLS (green) and nerve (red). PKM1 is highly expressed only in nerve areas, whereas PKM2 is expressed ubiquitously. Scale bars, 50 µm. Statistical analysis: unpaired two-sided t-test (a).
